# Physiology in conservation translocations

**DOI:** 10.1093/conphys/cou054

**Published:** 2014-12-17

**Authors:** Esther Tarszisz, Christopher R. Dickman, Adam J. Munn

**Affiliations:** 1 School of Biological Sciences, University of Wollongong, Wollongong, NSW 2522, Australia; 2 School of Biological Sciences, University of Sydney, Sydney, NSW 2006, Australia

**Keywords:** Conservation physiology, conservation translocation, monitoring, vertebrate

## Abstract

Conservation translocations aim to restore species to their indigenous ranges, protect populations from threats and/or reinstate ecosystem functions. They are particularly important for the conservation and management of rare and threatened species. Despite tremendous efforts and advancement in recent years, animal conservation translocations generally have variable success, and the reasons for this are often uncertain. We suggest that when little is known about the physiology and wellbeing of individuals either before or after release, it will be difficult to determine their likelihood of survival, and this could limit advancements in the science of translocations for conservation. In this regard, we argue that physiology offers novel approaches that could substantially improve translocations and associated practices. As a discipline, it is apparent that physiology may be undervalued, perhaps because of the invasive nature of some physiological measurement techniques (e.g. sampling body fluids, surgical implantation). We examined 232 publications that dealt with translocations of terrestrial vertebrates and aquatic mammals and, defining ‘success’ as high or low, determined how many of these studies explicitly incorporated physiological aspects into their protocols and monitoring. From this review, it is apparent that physiological evaluation before and after animal releases could progress and improve translocation/reintroduction successes. We propose a suite of physiological measures, in addition to animal health indices, for assisting conservation translocations over the short term and also for longer term post-release monitoring. Perhaps most importantly, we argue that the incorporation of physiological assessments of animals at all stages of translocation can have important welfare implications by helping to reduce the total number of animals used. Physiological indicators can also help to refine conservation translocation methods. These approaches fall under a new paradigm that we term ‘translocation physiology’ and represent an important sub-discipline within conservation physiology generally.

## Abstract (precis)

Translocations (reintroductions) are prominent in conservation, but successful outcomes remain few, despite advances in the field. Physiology is a hitherto underutilised, but could markedly improve successes. More importantly, increased application of physiological tools actively promotes at least two of the three Rs of animal welfare – Refinement of methods and Reduction of animals used.

## Introduction

The translocation, reintroduction and introduction of species to areas within their former range (or to areas considered appropriate or amenable to their survival and persistence) are entrenched and popular methods in conservation biology (Osborne and Seddon, 2012). These methods serve to improve the conservation status of focal species or restore ecosystem functions and processes (IUCN/SSC, 2013). Such deliberate transfers to promote conservation outcomes are collectively termed ‘conservation translocations’, and include any movement of animals (or plants) for conservation purposes (Osborne and Seddon, 2012; Seddon *et al.*, 2012; IUCN/SSC, 2013). These transfers can be classified further into population restorations and conservation introductions (Seddon*, et al.*, 2012; IUCN/SSC, 2013); see Table 1. Population restorations involve either reinforcement of existing populations by movement and release of conspecifics or reintroduction of extirpated animals into their indigenous range (IUCN/SSC, 2013). Conservation introductions involve moving organisms outside of their indigenous ranges either to avoid extinctions (i.e. assisted colonization; Thomas, 2011; Seddon *et al.*, 2012; IUCN/SSC, 2013) or because the organisms perform a specific function within the ecosystem, i.e. ecological replacement (Armstrong and Seddon, 2008; Seddon*, et al.*, 2012; Seddon and van Heezik, 2013; IUCN/SSC, 2013); examples of the latter species include ecosystem engineers and apex predators (Letnic *et al.*, 2012; Ritchie *et al.*, 2012; Seddon and van Heezik, 2013).

**Table 1:** Definitions of terms used in reintroduction projects (based on IUCN/SSC, 2013)

**Conservation translocation:** the intentional movement and release of a living organism where the primary objective is a conservation benefit
**Population restoration:** any conservation translocation within indigenous range. This comprises the following two activities:
(i) **reinforcement:** the intentional movement and release of an organism into an existing population of conspecifics; and
(ii) **reintroduction:** the intentional movement and relase of an organism inside its indigenous range, from which it has disappeared
**Conservation introduction:** the intentional movement and release of an organism outside its indigenous range. The following two types are recognized:
(i) **assisted colonization:** the intentional movement and release of an organism outside its indigenous range to avoid extinction of populations of the focal species; and
(ii) **ecological replacement**: the intentional movement and release of an organism outside its indigenous range to perform a specific ecological function

In recent years, there has been an exponential increase in the number of conservation translocation projects worldwide (Seddon *et al.*, 2007), and there have been several excellent reviews of reintroduction/translocation success in particular taxa (e.g. Griffiths and Pavajeau, 2008; Finlayson *et al.*, 2010) and of directions in the field more generally (Ewen *et al*., 2012a). However, despite this increase in conservation translocation research, much of this work has focused on more easily assessable aspects of translocation protocols, such as release techniques, or on readily measured demographic aspects, such as short-term survival rates. Consequently, less tractable but potentially critical aspects of the translocation process remain uncertain. One key factor that could significantly affect the success of translocations and improve protocols concerns the biology of individual animals, and specifically, their physiological state, both pre- and post-release. Without doubt, the wellbeing of individual animals in translocations is well considered by practitioners, but within the published literature it is apparent that animal physiology is often under-represented as a feature of direct concern. Deeper consideration of the physiology of individuals and populations from a conservation perspective falls within the domain of the emerging discipline of conservation physiology (Wikelski and Cooke, 2006; Chown and Gaston, 2008; Cooke and O'Connor, 2010).

To evaluate the potential for physiology to inform and enhance conservation and translocation science, we aim here to consider the factors that promote success in conservation translocations and to focus on the role that conservation physiology might play. Thus, our review builds on concepts addressed by Fischer and Lindenmayer (2000) and Seddon *et al*. (2007), but adds new dimensions that have been little addressed hitherto in the published literature. To focus the review, we consider only studies of terrestrial vertebrates and aquatic mammals; these groups dominate in translocation studies and therefore offer the greatest opportunity to explore the role of conservation physiology in improving translocation success. We note that comprehensive translocation planning typically incorporates aspects of species' natural history (Pereira and Wajntal, 1999; Ottewell *et al.*, 2014), resource and environmental requirements (Rittenhouse *et al*., 2008), as well as economic, social and cultural needs (e.g. Williams *et al.*, 2002). Here, we emphasize the evaluation of species' biological requirements as being imperative for the success of translocation programmes, with particular focus on physiology.

### Aims of the review

Our specific aims are as follows: (i) to review conservation translocation papers for the presence or absence of quantitatively assessed physiological parameters; (ii) to assess the outcomes of conservation translocation studies; and (iii) to identify future directions for conservation translocation biology, with an emphasis on the role of conservation physiology.

## Physiology in conservation translocations

Definitions of conservation physiology vary among practitioners, but most agree that the discipline investigates the physiological responses of organisms to anthropogenic threats and stressors that may contribute to declines in their populations (Wikelski and Cooke, 2006; Franklin, 2009; Seebacher and Franklin, 2012; Cooke *et al.*, 2013) and that it provides a link between ecological patterns and environmental change (Seebacher and Franklin, 2012; Cooke *et al.*, 2013). Much as the definitions of conservation translocation have evolved to their current state, conservation physiology also has broadened in scope to identify and resolve problems that exist in populations, with increased inclusiveness of all taxa. The discipline also seeks to expand to identify problems at levels of still broader interest to conservation practitioners, including species, communities and ecosystems (Cooke *et al*., 2013).

Physiology, when applied to conservation management of populations, provides vital data on the causal mechanisms that underlie current population problems (Carey, 2005; Wikelski and Cooke, 2006; Franklin, 2009) and also has the potential to illuminate previously neglected or concealed conservation issues (Chown and Gaston, 2008). Multiple factors influence conservation translocations, with interconnections between behaviour, physiology and ecology that can determine population survival (Tracy *et al.*, 2006). This complexity is well illustrated in trials on resource acquisition by desert tortoises, which show how physiological processes interact with animal ecology and behaviour and are integral to the assessment of conservation status (Tracy *et al.*, 2006; Drake *et al.*, 2012; Cooke *et al.*, 2013). In other examples, physiological approaches are being increasingly used to identify and reduce the effects of disease in population declines (Blaustein *et al.*, 2012), to increase the sustainability of fisheries management (Cooke *et al.*, 2012), to enhance understanding of seed dispersal by animals (Ruxton and Schaefer, 2012) and even to improve conservation policy (Cooke and O'Connor, 2010). The call for use of physiology in restoration ecology was given significant evaluation in a review (Cooke and Suski, 2008) largely in relationship to plant taxa and restoration of degraded habitats; however, mention of vertebrate taxa and incorporation of physiological assessment tools such as bio-monitoring, use of stable isotopes and doubly labelled water was called for, with a note of the increased convenience of these tools.

In terms of conservation science more generally, interest in conservation physiology arises because it offers an opportunity to predict the responses of organisms to environmental change (Carey, 2005; Wikelski and Cooke, 2006; Franklin, 2009; Kearney *et al.*, 2010; Seebacher and Franklin, 2012), thereby informing actions and policies that might improve conservation outcomes. With the current challenge of climate change and its potentially catastrophic impacts on biodiversity in many regions, the playing field for reintroduction biology has moved. As emphasized by leading texts and articles (e.g. Thomas, 2011; Osborne and Seddon, 2012; Bekoff, 2013), climate change has altered the context of conservation translocations because conditions often cannot be restored to ‘the way they were’; the original conditions simply no longer exist. Therefore, it is increasingly important to understand the physiological tolerances of vulnerable and endangered species in order to identify whether they have the physiological capability to adapt to changing climates or to respond to other anthropogenic modifications to the environment (Kearney and Porter, 2009; Smith, 2011).

It is apparent from these and other considerations that physiological data are important in the development of conservation protocols to improve rates of success in conservation translocations. This is particularly relevant with respect to understanding species' demographic performance and predicting the possible impacts of climate change and other environmental disturbances. Thus, we introduce the term ‘translocation physiology’ to describe the explicit evaluation of physiological parameters throughout the translocation process. This includes, but is not limited to, pre-release, the translocation event and post-release monitoring.

### Translocation physiology

The adoption of physiology generally into conservation is an implicit acknowledgement of a previous deficit in conservation practice, especially—as we contend here—in reintroduction biology. Translocations are generally acknowledged as unavoidably stressful events (Dickens *et al.*, 2010; Parker *et al.*, 2012; Seddon*, et al.*, 2012). The translocation itself is likely to be highly distressing, from capture and handling to transport to release (Dickens *et al.*, 2010; Parker *et al.*, 2012). In an elegant example of this, Waas *et al*. (1999) used simulated translocation events for red deer (*Cervus elaphus*; including catching/herding, pre- and post-transport confinement, loading on and off vehicles and road travel) and made detailed physiological evaluations of heart rate, haematocrit, cortisol and biochemical parameters, such as blood sodium, lactate, glucose and magnesium. Even after habituation of animals to the simulated translocation, the real event remained stressful. Animals showed consistently increased heart rates and concentrations of blood lactate and cortisol (Waas *et al.*, 1999); elevated cortisol or corticosterone, depending on species, is a typical response to physiological stress (Romero, 2004, Romero and Butler, 2007). Immediate post-release mortality can have significant impacts on the success of population establishment (Armstrong and Seddon, 2008; Armstrong and Reynolds, 2012; Parker *et al.*, 2012).

Understanding and minimizing animal stress in translocations is clearly important (Dickens *et al.*, 2010; Parker *et al.*, 2012), and current literature rightly recommends that appropriate husbandry and release techniques be considered alongside knowledge of the biology and ecology (abiotic and biotic requirements) of any individuals that are to be translocated (Parker *et al.*, 2012; IUCN/SSC, 2013). This is a key recommendation of the IUCN guidelines for translocations, and emphasizes further that understanding the physiological status of both individuals and populations is a necessary and vital component of the translocation process.

Physiology enables a more in-depth understanding of individuals, populations and communities and can assist in discerning potential responses of organisms to environmental change (Cooke *et al.*, 2013). As knowledge of physiology elucidates cause-and-effect relationships (Cooke *et al.*, 2013), its usefulness in pre- and post-translocation planning cannot be overstated. Translocation physiology can assist in all stages of the translocation process in the following ways: assessing the consequences of outbreeding and inbreeding depression; improving understanding of immune responses to captivity and release stressors and their consequences (e.g. fitness, disease expression); testing the suitability of habitats for populations; identifying threats that might cause success or failure; identifying optimal habitats; linking fitness of organisms to environmental conditions; and providing credibility and greater certainty about the process (Cooke *et al.*, 2013).

## Review of literature

For our review of conservation translocations, we separated research papers into four distinct categories: pre-release; conservation translocation; post-release; and reviews. ‘Pre-release’ denoted any study dealing only with preparation for a reintroduction and not the act of the reintroduction itself. ‘Conservation translocation’ denoted any study detailing the process and execution of one or more conservation translocation projects. ‘Post-release’ denoted any study that dealt with the events following a translocation, but not the event itself. ‘Reviews’ are self-explanatory. Conservation translocation papers alone were evaluated for their inclusion of physiological evaluation because neither the pre-release nor the post-release papers covered the translocation event; these were noted but not used in our attempt to review the physiological factors that were considered in primary works. Occasionally, a paper covered more than one category. For example, Van Manen *et al.* (2000) described a number of releases of red wolves (*Canis rufus*), as well as pre-release preparation and post-release information in what was almost a review of the subject. In these cases, if translocation events were presented with other information, the paper was considered a ‘conservation translocation’ study and not placed in other categories. To meet the first aim of our review, we then scored papers that had used physiology as part of their protocol as well as other factors, such as genetics, behaviour, habitat and whether key threatening processes had been considered in the translocation process (Table 1). A full list of papers evaluated is available online as supplementary material.

Our intention was not to obtain an exhaustive summary of every translocation publication in the last decade, but rather to collate papers that would provide an indication of general trends in the field. Due to the marked influence of the review by Fischer and Lindenmayer (2000), we carried out a detailed search for relevant studies in the same 12 international journals that were used in this earlier work. We focused on the years 2000–2010. These 12 journals, as well as *Trends in Ecology and Evolution*, were searched issue by issue for articles containing the words translocation, reintroduction or augmentation, and all papers concerning mammals, birds, reptiles and amphibians were considered (fish and invertebrates were beyond our scope). Using Google Scholar, we entered the same search terms as for our target journals and collated studies published in the 10 years up to 2010. We did not include studies that had not been peer reviewed, nor did we search for studies that had been cited in published papers but had been overlooked in Google Scholar. We assumed that our search methods were unbiased or at least not biased in any systematic way and that the years we reviewed provide a reasonable sample of recent reintroduction studies.

Rehabilitation does not fall under the definition of conservation translocation according to the current IUCN/SSC guidelines (IUCN/SSC, 2013) because the release is considered to be for the welfare of individual animals rather than for organizations at higher levels, such as populations. We did, nonetheless, include three exceptional rehabilitation studies that were population based and thereby fulfilled our criteria for adequate and quantitative reporting of reintroduction results (Goldsworthy *et al.*, 2000; Manire *et al.*, 2003; Molony *et al.*, 2006).

We acknowledge that published papers designed to answer specific questions may not be representative of entire translocation projects, as opposed to translocation proposals and reports that are submitted to conservation agencies, and thus there may be inherent difficulties in subjecting these to meta-analysis or other forms of quantitative review (D. Armstrong, personal communication). However, as peer-reviewed published literature is often the most readily accessible and primary source of background information on new translocation projects, we view the papers we examined as being broadly representative of the practices used currently by scientists involved in conservation translocations. To ensure the robustness of our approach and conclusions, we also consulted two influential recent works synthesizing current trends and past and present data on reintroduction and translocation biology (Ewen *et al.*, 2012a; Bekoff, 2013). We also consulted the most recent reintroduction guidelines provided by the IUCN (IUCN/SSC, 2013).

### Evaluation of success

With regard to assessment of the outcomes of conservation translocation studies (Aim ii), given that each project evaluated had its own definition of success and was carried out over a different time scale, we attempted to create specific criteria to determine the success of individual translocation projects in a repeatable and rigorous manner. We considered each study on its own merits. In the first instance, we evaluated success or otherwise of a translocation project based on each study's self-evaluation. However, some studies, while considering their project a success, failed to meet their stated aims or, in our reading of the results, failed to state reasonable reasons for considering the project a success. Therefore, in addition to self-reported success and failure, we introduced a binary category for projects deemed successful, this being to denote ‘high’ or ‘low’ success.

High success was determined if at least one of the following criteria was met.
  - The translocation confirmed that a stable and/or increasing population was established during the study period.
  - The project achieved its specified aims. For example, a project evaluating the effects of pre-release experience of elk (*Cervus elaphus*) with wolves (*Canis lupus*) and human hunters showed that experienced animals survived longer post-release, which was the specified aim (Frair *et al.*, 2007).
  - The project initially showed poor results, but improved them by altering protocols over time using information gleaned in earlier years (if releases took place over multiple years), i.e. there was some degree of adaptive management.
Low success was determined if at least one of the following criteria was met.
  - The study reported high success but failed to show conclusive results. For example, in a black bear (*Ursus americanus*) translocation that measured two different release techniques, >50% of study animals died or were unable to be included in the analyses due to lack of knowledge of their whereabouts (Eastridge and Clark, 2001).
  - A potentially threatening problem was present and could not be resolved, such as low genetic diversity due to small founder numbers or the presence of a key threatening process.
  - Catastrophic events occurred and significantly affected the project's results. For example, during the Iraq war the flight of Bedouins from Kuwait and Iraq to Jordan led to a doubling of the livestock population in the host country. This led to overgrazing, reduced water supplies and higher prevalence of disease and parasites in Jordanian habitats, compromising the translocation of oryx (*Oryx leucoryx*) as a result (Harding *et al.*, 2007).
  - The sample size was too limited to have resulted in a self-sustaining population as, for example, in the translocation of a single orang-utan (*Pongo abelii*) to Sumatra (Cocks and Bullo, 2008).
  - There was limited scope for population expansion and persistence. For example, despite the establishment of a reproducing population of lions (*Panthera leo*) in Phinda private game reserve, the population remained small and isolated, with little scope for connection to other isolated populations and for addressing the long-term conservation problems of the species (Hunter *et al.*, 2007).
  - The time of monitoring was too short to span even one breeding season. For example, a release of Pere David's deer (*Elaphurus davidianus*) in China spanned <6 months of monitoring (Hu and Jiang, 2002).

## Results

### Literature review

We reviewed 232 publications, of which 44 described pre-release protocols, 68 described post-release protocols and 120 reported conservation translocations, which are our primary focus below. The conservation translocation studies describe the translocation process in full, including pre-release factors, the translocation event itself and post-release monitoring. There were also 40 reviews. Traditional physiological factors were noted in 9% of the translocation studies. In comparison, 33% of the translocation studies considered genetics, 78% described behaviour and >80% considered habitat factors or key threatening processes associated with the translocation attempt (Table 2).

**Table 2:** Detailed breakdown of biological and environmental factors considered in 120 reintroductions of terrestrial vertebrates and aquatic mammals, showing numbers of projects rated as failures, successes and, in the latter category, high and low success

Biological or environmental factor	Total studies	Failures	Successes	Low success	High success
Genetics	39	3	36	15	21
Behaviour	93	12	81	32	49
Physiology					
Traditional physiology					
Stress physiology	3	1	2	1	1
Water, micronutrients	3	0	3	1	2
Thermoregulation	3	1	2	0	2
Immunoecology	2	1	1	1	0
Condition					
Distress	26	5	21	8	13
Body condition	46	5	41	13	28
Nutrition					
Wild food	12	0	12	5	7
Commercial food	11	5	6	2	4
Combination	19	1	18	5	13
Supplementary feeding	27	5	22	5	17
Other/unknown	18	1	17	8	9
Health					
Veterinary/health check	37	5	32	14	18
Vaccinations	7	1	6	5	1
Parasite management	15	3	12	6	6
Quarantine/disease screen	26	1	25	9	16
Unknown	2	1	1	0	1
Habitat					
Edge of former range	6	3	3	2	1
Core of former range	50	5	45	14	31
Combination of edge and core	1	0	1	0	1
Not reported	53	10	43	19	24
Predator-proof fence	9	0	9	3	6
Substitution	4	0	4	0	4
KTP					
Absent	49	3	46	14	32
Present	49	9	40	17	23
Unknown	22	5	17	7	10

See main text for definitions of ‘high’ and ‘low’ success.

### Physiology in conservation translocations

Detailed review of the 120 studies reporting conservation translocations suggested that physiological considerations could be broken down into four broad categories, i.e. condition, nutrition, health and ‘traditional’ physiology, each with two or more subcategories (Table 2). In total, 60% of studies (*n* = 72) reported the condition of animals that were being translocated and, of these, 86% were rated as successful (Table 2). Twenty-six studies (22%) noted whether animals showed distress reactions; 81% of these demonstrated success, with 62% of this subset rated as having highly successful outcomes (Table 2). Different approaches to assessing distress tended to be used on different vertebrate groups. For example, distress caused by handling and transportation was often considered in avian translocations, such as those involving the black-faced honeycreeper (*Melamprosops phaeosoma*; Groombridge *et al.*, 2004) and sharp-tailed grouse (*Tymphanchus phasianellus columbianus*; Coates *et al.*, 2006), and also in some involving mammals (e.g. red howler monkey, *Alouatta seniculus*; Richard-Hansen *et al.*, 2000). In these studies, researchers generally attempted to minimize the time that animals spent in transit, met their resource needs while they were being transported and ensured that benign weather conditions prevailed post-release. In contrast, while reactions to handling were mentioned in some projects that translocated reptiles, these ectotherms generally were considered to be most vulnerable to thermoregulatory distress. As such, housing during transit was usually the dominant factor that was considered as, for example, in a translocation study of the three-toed box turtle (*Terrapene carolina triunguis*; Rittenhouse *et al.*, 2008).

Body condition was used as an indicator of physiological state in 46 studies (38%), more frequently than any other physiological parameter. Although body condition may not be a direct measure of organism function, it is often assumed to correlate with individual ‘fitness’ (Marshall *et al.*, 1996), at least with regard to the ability of an animal to withstand potential stressors, such as immunological, nutritional or thermoregulatory challenges. Conservation translocation studies that considered body condition generally had high success; most used either qualitative indices of condition, such as visual appearance, or more invasive but direct estimates of body fat content (e.g. Woolnough *et al.*, 1997). Some studies also employed simple but quantitative indices based on regressions of body mass on linear measures of body size (e.g. body, limb or foot length; Krebs and Singleton, 1993; Schulte-Hostedde *et al.*, 2005). Here, relatively massive individuals lying above the regression line (i.e. with positive residuals) are considered to be in good condition and those below the line to be in poor condition. These residual-based indices of body condition need to be interpreted cautiously because body mass can fluctuate markedly over short periods, may not correlate well with other measures of body condition, such as body fat (Krebs and Singleton, 1993) and may vary as animals grow (Peig and Green, 2010). However, provided that these limitations are borne in mind, the high success of conservation translocation studies using residual-based indices (Table 2) suggests that this approach to judging condition has considerable utility.

Food and nutrition were evaluated in many translocation protocols (Table 2), with researchers providing food during the reintroduction process or as supplementary fare after animals had been released. All projects that fed animals natural or wild-type foods as part of their translocation (10%) were considered successful, with 58% of these deemed highly successful (Table 2). Studies where reintroduced animals were fed a combination of wild and commercial-type food (16%) had a similar high success rate of 95%, with 72% of these deemed highly successful, whereas those using only commercial-type food (9%) had a more mixed success rate of 54% (Table 2). Supplementary food after release was provided in 27 studies, generally as part of ‘soft’ release protocols that attempted to ensure that animals would not go hungry as they made the transition to eating naturally available foods (e.g. Richards and Short, 2003; Britt *et al.*, 2004; Brightsmith *et al.*, 2005). It is of note that 18 reintroduction studies provided food during the transfer or release stages but failed to specify the type of food offered or how it was provided. Despite these deficiencies in reporting, the overall results suggest that appropriate food is important during and after animals have been released and that success may be increased if natural foods are available to translocated animals before their release to the wild.

Using healthy animals would seem an obvious prerequisite for conservation translocation success (Stevenson and Woods, 2006), but health was mentioned in only half the studies we examined. Several studies advocated the need to make general heath checks prior to animals being released, both to maximize the survival chances of individuals and to minimize the potential for disease transfer to extant, resident populations of conspecific or congeneric species (Leighton, 2002; Mathews *et al.*, 2006).

‘Traditional’ physiological factors were considered in only 11 (9%) of the translocation studies reviewed (Table 2) and included assessments of stress using glucocorticoid hormone assays (Manire *et al.*, 2003; Pinter-Wollman *et al.*, 2009; Zidon *et al.*, 2009), as well as more direct evaluations of water use (Mathews *et al.*, 2006; Field *et al.*, 2007), micronutrient balance (Lapidge, 2005) and thermoregulation (Hardman and Moro, 2006; Rittenhouse *et al.*, 2008; Santos *et al.*, 2009). These studies were largely successful. Despite their emergence in other areas of wildlife ecology, such as in life-history studies (Martin *et al.*, 2006a, b), immunoecological approaches were used in only two of the translocation projects we evaluated. One study considered immunoecology tangentially by using the haematophil/lymphocyte ratio (see also heterophil/lymphocyte ratios) as an indicator of stress (Groombridge *et al.*, 2004), while the other used lymphocyte proliferation to evaluate immune function (Manire *et al.*, 2003). Haematological parameters were measured in a translocation study of the water vole (*Arvicola amphibius*, formerly *Arvicola terrestris*; Mathews *et al.*, 2006), but only erythrocytes were used to assess vole condition.

## Discussion

Conservation translocations and reintroduction biology are proceeding on a range of fronts, with varied protocols and different biological and environmental factors contributing to project success. In the sections below, we review some of the biases and weaknesses of conservation translocation projects, focusing particularly on physiology, and we identify some of the key design and methodological issues that influence the likelihood that a project will succeed.

### Translocation physiology: what can it offer?

The disciplines of behaviour, genetics and ecology are well-recognized elements in animal conservation biology and conservation translocation programmes, and their importance is clearly appreciated (Griffith *et al.*, 1989; Fischer and Lindenmayer, 2000; Letty *et al.*, 2007; Seddon*, et al.*, 2007; Groombridge *et al.*, 2012; Jamieson and Lacy, 2012; Keller *et al.*, 2012). However, a key disciplinary area that has received less attention in conservation translocation projects is that of physiology, especially those aspects of the discipline that can be considered relatively ‘traditional’ (Table 2). In this section, we focus on animal physiology in the pre-release and post-release design of conservation translocation projects and highlight how it can offer important insights to improve both initial and ongoing translocation success.

#### Pre-release planning

Setting *a priori* hypotheses provides opportunities to answer targeted questions concerning the species of interest, to test the importance of predefined factors that may influence translocation success and to distinguish the relative merits of different translocation protocols (Dickman, 1996; Armstrong and Seddon, 2008).

Recent literature on reintroduction and translocation biology (Ewen *et al.*, 2012a; Bekoff, 2013) emphasizes the need for more quantitative and rigorously assessable monitoring, which includes the planning or ‘risk-assessment’ phases. For example, when considering habitat suitability for a reintroduction it is easy to assume that historical locations indicate suitable habitat, but in fact this can be an erroneous and misleading indicator of habitat preferences (Osborne and Seddon, 2012). Furthermore, habitat does not encompass only vegetation, but should include all the biotic factors associated with it (Osborne and Seddon, 2012). Physiology has the ability to define cause-and-effect relationships and can therefore be used to adapt conservation management (Cooke *et al*., 2013). In terms of habitat, for example, physiological stress and condition parameters demonstrate how landscape patterns affect species persistence (Ellis *et al.*, 2012). Osborne and Seddon (2012) recognize that process-based species distribution modelling requires knowledge of physiological limits, but the authors also point out that ‘they are often not available’. As suites of physiological monitoring tools become more sophisticated, understanding of physiological limits should increase and, in turn, greatly enhance the conservation translocation process.

#### Release

The release phase of the translocation process has received the greatest physiological focus in peer-reviewed papers and in the current reintroduction literature (Parker *et al.*, 2012; Seddon and van Heezik, 2013). We feel that acknowledgement of the stress of translocation is crucial, but thus far only stress hormones have been examined widely. Quantitative analysis post-release of other physiological factors may give a more robust picture of the effects of translocation on animals. The importance of understanding an animal's basic ecology and biology is well recognized (IUCN/SSC, 2013), but the need for physiological indices is less well established. If the aim is to reduce potential stressors then it follows that first we must fully understand the extent of stress on translocated individuals by collecting physiological indices as baselines before, during and after the translocation process.

#### Post-release monitoring: establishment and persistence

In order to gauge outcomes of reintroductions, post-release monitoring is required. It therefore follows that the duration of post-release monitoring should be an important factor when considering success. The establishment of persistent and self-sustaining populations is one of the ultimate aims of conservation translocations (Parker *et al.*, 2012) and, as such, it is necessary to determine whether translocated animals can carry out the following: (i) establish initially; (ii) reproduce successfully; and (iii) persist long term at the translocation site (or at the least persist independently following release, even if they disperse to different locations). Despite this, much of the work we reviewed focused on assessing outcomes (i) and (ii), with few projects continuing to monitor for long enough to judge long-term establishment under outcome (iii). For example, most projects (72%) sustained monitoring for between 1 month and 5 years (see online supplementary material). This period is unlikely to cover more than a few generations for any vertebrate species and perhaps reflects other imperatives, such as the period over which interest or funding is available (e.g. many national and international funding schemes, such as the Australian Research Council, US National Science Foundation, provide grant funds for 2–5 years). Consequently, most projects that putatively demonstrated outcomes (i), (ii) and (iii), and thus self-evaluated as successful, were somewhat limited in their post-monitoring scope.

Current reintroduction literature (Ewen *et al.*, 2012a; Seddon and van Heezik, 2013) and the IUCN/SSC (2013) guidelines advise the following: pre-release baseline ecological data; demographic performance; behavioural monitoring; ecological monitoring; genetic monitoring; health and mortality monitoring; and social, cultural and economic monitoring. This is a comprehensive list, but we argue that the use of physiological indices to gauge both individual and population-level performance should be introduced explicitly. For example, acknowledgement that physiological differences and tolerances in and between individuals can affect population diversity (Cooke *et al*., 2013) has broad implications for long-term translocation success. Notably, health monitoring and conservation medicine are well established and fundamental to reintroduction biology (Aguirre, 2002), but we suggest that non-clinical, pre-clinical and peri-clinical physiological aspects of individuals' biology could further advance the field of conservation translocations

### Translocation physiology: promoting two of the three Rs of animal welfare

The three Rs of animal welfare and ethics in research are well-established doctrines that promote the replacement (R1), reduction (R2) and refinement (R3) of animals used for research. These are highlighted as key considerations for any activity relating to animal research and necessarily extend to conservation and reintroduction biology. However, despite tremendous advances in the science of reintroduction biology (Ewen *et al.*, 2012a; Seddon and van Heezik, 2013), there remains a ‘more animals’ approach to reintroductions/translocations, at least tacitly by some conservation practitioners, in the hope that some animals will survive and establish self-sustaining populations. This is not to suggest that the ‘more animals’ approach reflects active intentions or a lack of consideration for animal welfare and wellbeing, nor the view that ‘more animals’ is the best option for success, but it probably reflects the simple consequence of having the opportunity to release large numbers of animals, combined with low expectation for survival, presumably because information about how the animals will be impacted by release is necessarily limited. Nonetheless, we argue that this approach contravenes R2 and R3 of the codes of practice and recommendations from national and international animal ethics and welfare bodies.

Obviously, replacing animals (R1) for reintroduction is not possible, but the incorporation of physiology and physiological measures into the translocation paradigm could markedly improve the survival chances of released animals, as well as improving our understanding of the reintroduction/translocation process generally. These outcomes directly assist the principles of reducing the total number of animals (R2) and the refinement of methods (R3) to promote successful reintroductions and translocations. By extension, this also serves to achieve R1 (replacement of animals) by ultimately obviating the need to reintroduce further animals once a population has become self-sustaining. This last point is not trivial, in that once a self-sustaining population is established, further monitoring of animals and their habitat and ecosystem more generally should then become a key aim of management, with the aim of eliminating further need for captive rearing and release or translocation.

From a practical perspective, the ‘more animals’ approach can also be fiscally irresponsible, because of the generally high costs associated with rearing and releasing large numbers of animals. Many conservation and reintroduction organizations rely heavily on public support as charity, in addition to the financial support of government and non-government research organizations. As such, it is imperative that animals are used only when the chances of translocation success can be demonstrated to be high and that every action has been examined and evaluated with a view to maximizing the likelihood of success of establishment of self-sustaining populations.

Given the inherent invasiveness of reintroductions generally, we argue that it is necessary to consider whether invasive and non-invasive physiological procedures should be given more consideration than has occurred to date. Translocations should be not only cost-effective, but also ethical undertakings, in that only the minimal numbers of animals needed to ensure success are used. The idea of releasing large numbers of animals in the hope of having a few survive is, in our view, unacceptable, particularly given recent advances in conservation physiology that can help to improve the efficiency of breeding and reintroduction programmes. We consider some of the most relevant advances below.

## Physiology and conservation translocation

### ‘Stress’ in conservation translocations

‘Stress’ consists of three interrelated components: stressors, which are the environmental stimuli that lead to a stress response; acute stress; and chronic stress (Romero and Butler, 2007). Translocations often involve multiple stressors, each of which can activate acute and longer lasting responses (Dickens *et al.*, 2010; Parker*, et al.*, 2012). Typically, a stress response begins with an immediate adrenocorticoid (fight-or-flight) cascade, characterized by the production of glucocorticoids or ‘stress hormones’ (Romero, 2004; for detailed descriptions of the endocrinological processes involved in stress see also: Romero and Butler, 2007; Dickens *et al*., 2010; Parker *et al*., 2012). Therefore, the easiest and most common indicator of animal stress that could be monitored in translocation is the glucocorticoid response (Manire *et al.*, 2003; Hartup *et al.*, 2005; Pinter-Wollman *et al.*, 2009; Zidon *et al.*, 2009). The main glucocorticoids used in wildlife studies are cortisol (many mammals) and corticosterone (rodents, birds, amphibians and reptiles); their roles in stress and as measures of stress have been reviewed extensively (Romero, 2004; Romero and Butler, 2007; Dickens *et al.*, 2010; Parker *et al.*, 2012). Glucocorticoid production can persist as part of a longer term response to stressors (Romero and Butler, 2007), and its major effects include behaviour modification, increased blood glucose levels, inhibition of normal growth and reproduction, and depression of immune function (Romero and Butler, 2007). Additionally, for translocated animals, stress hormones may have unique and unforeseen impacts.

It is well known that glucocorticoids can affect almost all cell types and tissues (Dhabhar, 2009), and the changes they induce can be critically important for aiding survival and ameliorating recovery following distress. However, for naïve animals released into unfamiliar environments, as occurs during translocations, unusual or novel stressors may be particularly disruptive because naïve animals may have no behavioural or physiological frame of reference for displaying appropriate responses (Waas *et al.*, 1999; Romero, 2004; Dickens *et al.*, 2010; Rensel and Schoech, 2011). Consequently, the impact of novel stressors on translocated animals may be more severe and persistent than expected, with implications for the development and assessment of conservation translocation protocols.

Despite the benefits of acute or immediate responses to stressors, persistent or chronic exposure to stressors (or the perception of stressors) can have a range of deleterious effects (Millspaugh and Washburn, 2004; Dhabhar, 2009). Persistent distress, for example, can impair feeding behaviours, thereby compromising daily energy and nutrient acquisition; it can also increase energy requirements (Dickens *et al*., 2010), thus presenting animals with conflicting challenges. Additionally, persistent endocrinological responses to stressors can dampen the immune systems of animals, depressing their abilities to respond to immune challenges (Dhabhar *et al.*, 1996), such as injury or exposure to pathogens or parasites (Bortolotti *et al.*, 2009). Such challenges can further stimulate stress responses, leading to synergistic cascades that may increase risks from further immune challenges (Woodford, 2002). These compounding problems are likely to be important for translocated animals because new environments may also expose them to new or different strains of pathogens and parasites and may be particularly problematic for captive-born and-reared animals that have had limited or no prior pathogenic exposure. In this regard, captive-born and-raised animals present a particular conundrum with regard to innate immunity and host–parasite interactions, simply because they may lack the acquired immunity associated with prior exposure (Mathews *et al.*, 2006; Ewen *et al.*, 2012b). Thus, at the very least, pre-release health checks and vaccinations for appropriate diseases should be considered highly desirable, but we suggest also that breeding and release projects consider ‘training’ animal immune systems through direct challenges during the rearing process.

As the main components of translocation—capture, captivity, transport and release into a novel area—are all individually stressful events (Parker *et al.*, 2012), translocated animals will inevitably experience some degree of acute and/or chronic stress. This can lead to changes both in stress response physiology (fight-or-flight responsiveness, sympathetic nervous system drivers, hypothalamic–pituitary–adrenal axis function and overall glucocorticoid secretion) and in the function of the immune system and behavioural coping strategies (Dickens *et al.*, 2010).

Stress may not be a frequent or direct cause of translocation failures, but it can certainly jeopardize the principal objective of most release projects, that being to establish self-sustaining populations. In this regard, chronic or persistent exposure to stressors is important because it can disrupt animal reproduction, both endocrinologically (Sapolsky *et al.*, 2000; Berga, 2008) and behaviourally (Romero and Butler, 2007). Persistent stress responses by translocated animals can potentially be disastrous for the relevant species and for the specific release project (which may also jeopardize future funding prospects). Consequently, given the potential for translocations to perpetuate cycles of persistent stress, immune compromise and reproductive failure, we argue that ongoing monitoring for indications of stress should be incorporated explicitly into conservation translocation protocols. Techniques for such monitoring may involve the invasive sampling of tissue or body fluids, such as blood or saliva, or the non-invasive collection of waste or shed material, such as hair or feathers (Table 3), and thus may be selected as appropriate to the species that is being translocated.

**Table 3:** Physiology in the field: invasive and non-invasive measurements that can be made to help facilitate success in conservation-based reintroductions of animals

Physiological measurement	Biological material or method	Invasive or non-invasive	Examples
Glucocorticoid ‘stress’ hormones			
	Blood	I	McKenzie *et al*. (2004)
	Saliva	I	Pearson *et al*. (2008)
	Faeces	NI	Hartup *et al*. (2005)
	Urine	NI	Sheriff *et al.* (2011)
	Hair and feathers	NI	Bortolotti *et al.* (2009)
Thyroid hormones			
	Blood	I	Yochem *et al.* (2008)
	Faeces	NI	Wasser *et al.* (2010)
Reproductive hormones			
	Blood	I	Brown (2000)
	Faeces	NI	Wasser and Hunt (2005)
	Urine	NI	Graham (2004)
Trace elements			
	Blood	I	Lapidge (2005)
Stable isotopes			
	Blood	I	Janssen *et al*. (2011)
	Faeces	NI	Varo and Amat (2008)
	Hair and feathers	NI	Cerling *et al*. (2006)
Bio-monitoring (e.g. heart rate, temperature)			
	Implants	I	Waas *et al*. (1999)
	Remote sensing	NI	Lavers *et al.* (2009)
Metabolic rate and water turnover	Labelled water	I	Lapidge and Munn (2012)

Abbreviations: I, invasive; and NI, non-invasive.

### Beyond ‘stress’: other useful physiological indicators

#### Health indices

Several field-based measurements can be used as indicators of the general health and wellbeing of individual animals or populations (Tables 2 and 3). It is important to identify which measures and methods (especially invasive *versus* non-invasive methods; see Table 3) will be most appropriate for particular species. Selection will depend on a range of factors, including the target animal's body size and life history, the degree of association that individuals have had with people and the ease of sample collection and storage. Other factors may also need to be considered for specific translocations, such as whether animals will be translocated most effectively while conscious or immobilized and, if the latter, whether appropriate anaesthetic drugs and personnel trained to administer these will be available.

Health and immunocompetence underpin the survival of individual animals but may also provide insights into the health of populations more broadly. Poor health, for example, increases the risk of depredation (Krumm *et al.*, 2010) and can lower reproductive success (Cook *et al.*, 2004); each of these deficits is especially important in the context of conservation translocations because of the often small number of founder animals released and because even small losses or reproductive impairments are likely to have major deleterious effects on project success. Basic pre-translocation evaluations of individual health have contributed to the success of captive-bred chimpanzees released into the Conkouati Reserve (Tutin *et al.*, 2001) and to translocations of water voles (Mathews *et al.*, 2006) and bighorn sheep (Ostermann *et al.*, 2001), but health assessments rarely extend beyond the release period.

The potential to transfer pathogens and parasites endemic in one location to a new location is another health-related concern relevant for animal translocations and, to a lesser extent, for captive-bred releases (Ewen *et al.*, 2012b). Importantly, when considered solely from a veterinary or health-evaluation perspective, the fact that an organism is non-pathogenic in one area may overlook the risks that pathogens or parasites could become problematic for animals moved to a new site (Armstrong and Seddon, 2008; also see Mathews *et al*., 2006 for a detailed discussion on the health of translocated water voles and captive dibblers, *Parantechinus apicalis*). Conversely, transmission of a disease from a hitherto unknown reservoir at a release site can also occur. For example, reintroduced African wild dogs (*Lycaon pictus*) contracted rabies after ingesting infected jackal carcasses, despite the wild dogs being vaccinated for rabies pre-release (Woodroffe and Ginsberg, 1999). Such vulnerabilities may be particularly important for captive-bred animals, which have vastly different life experiences in comparison to wild-caught animals used for translocation. Overall, efforts to establish health status and the immunocompetence of animals to be translocated could have profound benefits for conservation translocations. As such, key indicators of animal health status that are easy to access and track pre- and post-release could prove exceptionally useful in the translocation biologist's ‘tool box’. We suggest below that thyroid hormones are good candidates for such health-tracking markers and may offer tangible benefits for translocation projects generally.

#### Thyroid hormones

Thyroid hormones [thyroxine (T_4_) and triiodothyronine (T_3_)] convey important information about overall health and disease status in animals (Yochem *et al.*, 2008), and they can also provide insight into an animal's underlying metabolic state (Rolland, 2000; Wasser *et al.*, 2010) and thermoregulatory capacity. Additionally, thyroid hormones convey information about growth and development, including brain development (Silva, 2006; Wasser *et al.*, 2010). Thus, characterization of the thyroid status of individuals or groups of animals could contribute substantially to our understanding of their general health and wellbeing. Perhaps more importantly, measures of animal thyroid status could also identify sub-clinical (or undiagnosed clinical) diseases or other maladies (Mönig *et al.*, 1999; Mooney *et al.*, 2008) that may not be evident from cursory observations of animals. Maintenance of peak health is likely to be vital during all stages of a reintroduction procedure, from animal release to survival post-release, and to successful reproduction and population establishment. Hence, the assessment of animals' thyroid hormone status, accessed invasively or non-invasively (see Table 3), can offer an important indicator of health and survival prospects as well as overall population viability. We suggest also that ongoing or even *ad hoc* evaluations of the thyroid status of translocated animals may highlight hitherto unknown or unforeseen interactions between animal health, survival and ecology, thereby improving the science and the success of animal translocations more broadly.

#### Nutritional physiology

Many studies in our review evaluated habitat characteristics with a view to ensuring that adequate food resources would be available to animals post-release. However, most studies also assumed that habitat equated to food resources and overlooked important interactions between animal physiology and nutrition (but see Lapidge and Munn, 2012). The finding that critical food items are apparently available is not necessarily a reliable indication of how well an animal can access or use the resources appropriately. For example, there may be physical, behavioural or ecological constraints (e.g. the presence of other species) that preclude individuals from accessing food (e.g. Dickman, 1991). The role of nutritional physiology is perhaps the most neglected aspect of translocation biology, perhaps because it is not easily assessed. However, some methods are tractable and also readily accessible for conservation translocation programmes.

Nutritional physiology encompasses more than a simple accounting of the foodstuffs that are available at a release site, and potentially considers a wide range of factors that are relevant to translocations. These factors include the phenotypic plasticity of the gastrointestinal system (Starck, 1999a, b, 2005; Millán *et al.*, 2003; O'Regan and Kitchener, 2005; Starck and Wang, 2005; Munn *et al.*, 2006, 2009), the impacts of gut pathogens (Everest, 2007), microbes or other intestinal symbionts that are needed for healthy digestion (Hooper and Gordon, 2001; Kohl and Dearing, 2012), and microbial ‘seeding’ of captive-reared animals, particularly herbivores, to aid digestion following release, and even foraging behaviours; all of these factors can ultimately affect survival and breeding success.

Ensuring nutritional and digestive wellbeing may be critically important for captive-bred animals, especially if they have been reared on highly processed or commercial foods. Often, captive-bred animals do not have to ‘work’ for their food, at least not as intensively as their wild counterparts. As such, there are likely to be significant interactions between the nutritional experience of captive-reared animals and how they fare following release. Specific studies of these interactions are rare, but they could be investigated empirically using soft- and hard-release methods where animal condition can be observed. For example, in a study of released Peninsular bighorn sheep (*Ovis canadensis*), all released animals were fed on high-quality food (alfalfa pellets plus salt and mineral blocks) in addition to having access to native vegetation in pre-release enclosures (Ostermann *et al.*, 2001). The animals were then released into the wild without immediate acclimitization to a diet consisting solely of native vegetation. The project failed to establish a self-sustaining population (Ostermann *et al.*, 2001) and, although numerous explanations were offered to account for the poor success, we contend that nutritional physiology was likely to have been relevant; indeed, the authors themselves suggested that higher success in certain releases was related to the availability of good-quality forage and water (Ostermann *et al.*, 2001).

It is apparent that abrupt dietary changes can generate negative outcomes for animals by increasing stress and depriving them of key nutrients, both of which may lead to compromised immunity immediately post-release. The gastrointestinal tract is keenly influenced by the immune system, where the immune cells and resident microbes form a complex ecosystem (McCracken and Lorenz, 2001). This intestinal ecosystem can be altered by changes in diet (Liukkonen-Anttila *et al.*, 2000; McCracken and Lorenz, 2001) and can further influence other physiological features, particularly when animal stress hormones are elevated (Everest, 2007). Recent studies of wild vs. captive wood grouse (*Tetrao urogallus*; Wienemann *et al.*, 2011), for example, have revealed major differences between the gastrointestinal microbiota of wild and captive birds. In the context of translocation biology, mismatch between the appropriate intestinal environment and that established in the released animals could adversely affect the survival of translocated animals. In another study, marbled teal (*Marmaronetta angustirostris*) maintained for a longer captive period before release showed lower survival rates compared with those released soon after fledging, and this was attributed to the longer held animals being fed a commercial diet (Green *et al.*, 2005). Therefore, dietary adjustments should be considered thoroughly in translocation protocols and, given that gut flexibility (both in terms of morphology and microbial composition) takes time to adjust (e.g. Moore and Battley, 2006), a gradual reduction of high-quality foodstuffs prior to release may improve survival post-release.

Assessment of micronutrients and trace elements is another component of nutritional physiology that holds potential value to translocation physiology. This is especially the case with respect to releases of captive animals, as demonstrated by Lapidge (2005). In that study, plasma vitamin E concentration was evaluated in yellow-footed rock wallabies (*Petrogale xanthopus celeris*), due to prevalence of deficiencies in captive but not wild animals (Lapidge, 2005). The study aimed to assess the welfare implications of releasing captive wallabies and demonstrated how the captive animals adjusted to the wild environment by rapidly increasing plasma vitamin E concentrations post-release to levels similar to their wild counterparts, thus indicating that there were no appreciable welfare implications.

Overall, nutrition is one of the more easily manipulated aspects of the translocation process and potentially also one of the most important. Nutrition can be manipulated non-invasively and with little expense, and the benefits of incorporating nutritional aspects of physiology should have flow-on effects for improved immune status, reproductive success and general animal health and wellbeing. For these reasons, we argue that more focus should be placed on priming the gastrointestinal tract of captive-reared animals before release and that additional factors, such as seasonal or diet-related plasticity of the gastrointestinal tract (Piersma and Lindström, 1997), should be incorporated into release protocols.

#### Other physiological factors

There is a collection of other physiological factors that could be of use to translocation physiology. Immunoecology (or ecological immunology) investigates underlying causes of immune system function between individuals and populations (Hawley and Altizer, 2011) and, as such, has close ties with health indices, disease and stress. Groombridge *et al*. (2004) demonstrated this via quantative evaluation of white blood cell counts to measure stress levels in Po'ouli (*Melamprosops phaeosoma*). Integration of immunoecological aspects of animal biology and techniques used to evaluate immune status in the wild may be particularly useful for understanding the cause-and-effect nature of translocation successes and failures.

Understanding a species' reproductive biology is also important for predicting the viability of wildlife populations, as well as for developing best practice captive-breeding programmes (Brown, 2000; Graham, 2004; Wasser and Hunt, 2005; Asa, 2010). Details of the reproductive physiology and associated needs (e.g. specific resources) have scope for further inclusion in managing translocated populations.

Stable isotopes can be used to study diverse factors affecting wildlife, all of which are relevant to conservation translocations. These can range from, for example, identifying factors that affect growth (Janssen *et al.*, 2011), determining migration patterns and diet changes (Cerling *et al.*, 2006) and teasing out species differences in dietary assimilation to determining why species with similar ecologies are displaying different survivabilities in the same habitats (Varo and Amat, 2008) and range from invasive to non-invasive techniques (Table 3).

The biology of stable and radioactive isotopes can also inform translocation science. Analysis of metabolic rate and water turnover can be used to measure how translocated animals, particularly those that are captive bred, adjust to wild conditions post-release, and can be a particularly sensitive measure of success, as demonstrated by Lapidge and Munn (2012).

### Translocation physiology: methods

Perhaps the most important aspect to consider prior to a translocation is whether invasive methods for monitoring physiology are appropriate, acceptable and practicable for the given situation. The level of information generated from physiological investigations should be expected to justify their use or to rank whether relatively less-invasive methods would be better suited to the species in question.

Non-invasive methods for monitoring animal physiology have two main benefits for conservation translocation biologists. Firstly, they minimize direct contact with animals, and secondly, they can minimize direct or remote exposure of animals to humans (Table 3). However, it is important to remember that translocation is, by its nature, an invasive procedure. Animals are captured (whether free-living or captive) and transported, usually to new and unfamiliar environments. The potentially profound impacts of translocation are highlighted by the often high mortalities that are seen for newly released animals. In a study of reintroduced European mink (*Mustela lutreola*), for example, mortality exceeded 40% in the first 30 days post-release (Maran *et al.*, 2009). In a translocation of radio-collared elk (*Cervus elephas*), 15% of deaths occurred in the 6 weeks following release and were related to stresses associated with capture and/or release (Larkin *et al.*, 2003). Consequently, careful attention to physiological measures indicating animal distress or compromised health and wellbeing should be included explicitly in translocation protocols. For example, identification of key trigger points to initiate intervention during capture, transport and post-release could be crucial for ameliorating the apparently widely accepted high levels of post-release mortality in translocations. In particular, we suggest that a ‘more animals’ approach to combating the high rates of post-release mortality in conservation translocations may be less successful than a ‘fewer animals–more invasive’ approach.

The ‘more animals’ approach is problematic for several reasons, not least because it contravenes codes of practice and recommendations from national and international animal ethics and welfare bodies, which strive to reduce the numbers of animals used for science and research and to refine the methods used to maximize the success of animal-based projects. In addition, a ‘more animals’ approach is not fiscally responsible because of the generally high costs associated with rearing and releasing large numbers of animals. Therefore, given the inherent invasiveness of translocations, it is prudent to consider whether invasive procedures should be considered more often than has occurred previously, especially if this results in improved conservation translocation outcomes.

There are several invasive procedures that would probably benefit conservation translocation projects (Table 3) and that are appropriate for a range of taxa, including reptiles, mammals and birds. Of note, most of these procedures are well established in veterinary and physiological practice, making their inclusion in conservation translocation protocols relatively straightforward, especially if relevant experts are consulted. In this context, we suggest that several aspects of research could prove valuable for understanding and evaluating the entire translocation process, along with the mechanisms and factors that affect survival post-release. In particular, field metabolism (Lapidge and Munn, 2012), water use, heart rates and body temperature (Waas *et al.*, 1999) could be used to determine how well animals are acclimatizing or adapting to their new environments, whether they are maintaining condition, are foraging successfully and are able to meet the energetic and nutritional demands of reproduction. These are important questions, for which we have very limited data.

Radio- or GPS-tracking devices represent one semi-invasive method for evaluating animals post-release that has great potential for improving reintroduction success. Tracking devices can be considered invasive, in that they require animals to wear electronic tags, either externally (e.g. as neck or leg collars) or as internal implants. Such devices could interfere with animals' daily activities, but may also provide unprecedented information about how individuals adapt to release. For example, tracking can provide information on daily ranging patterns (Campioni *et al.*, 2013), insight into immediate post-release behaviours (Dennis and Shan, 2012) and otherwise cryptic, but critically important information about movements, habitats or nutrients that are essential for animal survival (e.g. Gurarie *et al.*, 2011). The ability to locate animals can assist with regular visual contact of subjects, thus allowing intensive behavioural monitoring, and can also present opportunities to collect additional physiological and behavioural information via collection of scats (providing information on, for example, diet and stress hormones) and urine (providing information on diet, stress hormones and water turnover). At the outset, placement of collars may require animals to be sedated, particularly for large mammals (e.g. Wear *et al.*, 2005), but this also provides an opportunity for collection of a wide array of baseline physiological data and indicators of animal health before release. Moreover, depending on the species and the situation, animals may be recaptured to replace the collar batteries or to retrieve GPS data, providing another opportunity to collect more invasive data, such as blood samples.

## Conclusions and recommendations

The weight heretofore given to genetic (Groombridge *et al.*, 2012; Jamieson and Lacy, 2012; Keller *et al.*, 2012), disease (Sainsbury *et al.*, 2012) and behavioural factors (e.g. Armstrong *et al.*, 1999; Ostro *et al.*, 1999; Munkwitz *et al.*, 2005) in translocation planning needs to be extended to include physiological processes and mechanisms as a recognized complementary discipline. Some resistance might be expected in promoting physiology as a critical tool for use in translocation biology. The view that physiological methods may cause distress, particularly for invasive methods like surgical implantation of heart rate monitors, has probably impeded the advancement of physiology in conservation science generally. Obviously, the potential use of physiological tools, their invasiveness and possible impacts must be weighed against the potential benefits to the survival of a given species or population, with the rarity of a species probably dictating the outcomes of these evaluations. Nonetheless, we argue that the role of physiology in reintroduction and translocation science should be given greater consideration. The most recent IUCN Guidelines for conservation translocations recognize that physiology should be assessed, and we echo that recommendation. In fact, we would go further, and argue that physiology is the principal unifier that describes the basic ecological and behavioural features of organisms relevant for evaluating any reintroduction proposal. To this end, we propose the following recommendations for developing and evaluating reintroduction projects.

- Reintroduction programmes should consider the range of interactions between released animals and the environment, including potential interactions with other species that may be present at the release site and that can be illustrated by invasive or non-invasive physiological indices. This should include, for example, the potential physiological responses to predators, competitors, parasites and pathogens. The potential for such interactions must be considered pre- and post-release and in follow-up monitoring studies, and mitigated if required.
- Databases of the physiology of reintroduced animals should be created prior to release, and they should include—at a minimum—information on genetic, behavioural, nutritional and health/disease aspects of the individuals being used.
- Greater use and consideration of physiological assessments of animal wellbeing pre- and post-release must be incorporated into monitoring protocols. This should assist in ensuring the suitability of animals for release and their performance thereafter. It will also become increasingly important to understand the physiological tolerances of reintroduced animals and species in order to predict their ability to adapt to changing conditions.
- Post-release monitoring should continue over longer periods than has been the case in most studies to date, particularly as conditions at many reintroduction sites are likely to change rapidly in future as the climate changes (Parmesan, 2006). Long-term monitoring is often not possible because typical funding cycles run for merely 3–5 years. Nonetheless, we urge that due consideration be given to defining and prescribing appropriate monitoring periods for specific reintroductions, partly to improve successes, but also to provide more realistic and rigorous evaluations of success. Moreover, monitoring of animal health and physiology should be considered at both early and later stages of reintroductions, either during or following acclimatization in ‘soft-release’ studies, and also over longer periods.

In conclusion, we note that substantive advances have been made in improving the success of animal reintroductions in recent years (Ewen *et al.*, 2012a). These advances have been assisted and supported by increased use of behavioural observations and ecological and genetic monitoring of released animals. However, from our review we argue that further advances in the field and in the success of individual reintroductions and translocations could be gained by broadening routine data collection to include relevant physiological measures. Such measures can inform researchers of the wellbeing of individuals and their chances of reproductive success and, thereby, the likelihood of a reintroduced population persisting post-release. As a starting point, we recommend that key indicators of animal health, such as cortisol and thyroid status, and of physiological state (e.g. condition, diet) be incorporated into routine pre- and post-release monitoring protocols. This is not to say that translocations or reintroductions should apply each of these recommendations unnecessarily, but they ought to be considered during planning for species-specific protocols, with a view to incorporating procedures strategically and in a manner most likely to benefit the success of the release. Nonetheless, given the persistent variability in the success rates of translocation, the collection of as many data as possible may assist future practitioners by accumulating a knowledge base of physiological indicators relevant to animal survival. Such indicators will help to identify potential problems that may not be apparent through *ad hoc* observations and offer the opportunity to improve translocations generally by focusing evaluations of ‘success’ on physiological wellbeing.

## Supplementary material

Supplementary material is available at *Conservation Physiology* online.

## Funding

No specific funding was awarded for this project. Esther Tarszisz is funded by the Australian Postgraduate Award (APA) scheme.

## Supplementary Material

Supplementary Data
