# Supplementary material for: Physiology in conservation translocations
Source: Conserv Physiol. 2014 Dec 17;2(1):cou054. doi: 10.1093/conphys/cou054 (PMC4732500; doi:10.1093/conphys/cou054)
Supplement: Supplementary Data [file supp_2_1_cou054__index.html]

Physiology in conservation translocations — Supplementary Data 

# Physiology in conservation translocations

## Supplementary Data

Supplementary Data

**Files in this Data Supplement:**

- Supplementary Data - Docx file
- Supplementary material references - docx file
