# Supplementary material for: Physiology in conservation translocations
Source: Conserv Physiol. 2014 Dec 17;2(1):cou054. doi: 10.1093/conphys/cou054 (PMC4732500; doi:10.1093/conphys/cou054)
Supplement: Supplementary Data [file supp_cou054_cou054supp.docx]

**Tarszisz et al**

**Supplementary material**

**S1: Criteria for data inclusion summarised in manuscript Table 2**

**(1) Criteria for inclusion**

Of the papers discovered in the literature search, only those with a conservation basis were considered. Studies without a conservation basis, such as those describing the relocation of nuisance urban animals (human-wildlife conflict), were excluded (see Massei *et al*., 2010 for a review of such studies), as were those that dealt with the translocation of wolves or other large predators as a non-lethal means of reducing predation on livestock ([Bradley *et al*., 2005](#_ENREF_11)). Studies that concentrated only on the source population, rather than the reintroduced population, were also omitted, e.g. ([Bain & French, 2009](#_ENREF_3)). Papers lacking sufficient information to be included in quantitative analyses were excluded, as were those that focused on specific aspects of the reintroduction process, such as how to improve the detection of the study species ([Reindl-Thompson *et al*., 2006](#_ENREF_101)). Papers that focused solely on anthropogenic factors involved in translocations (particularly those concerning carnivores) also were excluded if they lacked quantitative information on the reintroduction subjects themselves. For example, [Williams *et al*., (2002](#_ENREF_139)) described community attitudes to wolf (*Canis lupus*) reintroductions; this study was not included because it provided little quantitative insight into how wolves were reintroduced.

**(2) Analysis of the literature**

The search process yielded 232 papers (not including reviews) that met our criteria for inclusion. A full list of these papers is available in supplementary material. To examine broad trends in reintroduction research, we first considered the geographical regions where studies had been carried out and the species and taxonomic groups that had been studied. We then focused on the papers that we had classified as *reintroduction* studies and examined these in more detail. The following questions were asked:

1. What was the stated purpose of the study?
2. What period were releases carried out over?
3. Was any pre-release preparation initiated and, if so, what was the focus of this preparation (e.g. population viability analysis, habitat surveys)?
4. Was there any post-release monitoring and, if so, what was the nature of this (e.g. radio/GPS tracking, re-capturing, visual observation)?
5. How many animals were used?
6. Was the reintroduction considered a success or failure and, if deemed to be successful, was it of high or limited success? We define levels of success in the following section.

In addition to these questions, we defined a number of key biological and environmental factors that appeared likely to influence reintroduction success and asked whether these had been considered in the protocols of each study. These were scored as ‘yes’ or ‘no’ depending on whether the studies had met specific criteria for each factor. The criteria were as follows:

*(a) Genetics*

To receive a ‘yes’ for this category the study needed to have considered one or more of the following:

1. Number of founders and the effects this might have on inbreeding and/or outbreeding depression; and
2. Relatedness of individuals involved in the translocation (e.g. stock book, laboratory study of tissue samples for genetic analysis).

*(b) Behaviour*

To receive a ‘yes’ for this category the study needed to have considered one or more of the following:

1. Behavioural acclimation of animals to a new location (this was of particular importance for captive-born/raised animals);
2. Interactions with conspecifics; and
3. Predator/prey interactions (if relevant).

We considered the relative merits and disadvantages of soft- *versus* hard-release (i.e. releases, respectively, where supplementary food or shelter resources are, or are not, provided) to be part of pre-release preparation unless specific behavioural training was undertaken, the latter being a more directed and deliberate method typically aimed at improving success. Shier (2006) and [Shier & Owings (2006](#_ENREF_91)), for example, assessed the effect of predator training on the behaviour and post-release survival of captive prairie dogs (*Cynomys ludovicianus*).

*(c) Physiology*

This was further divided into four subcategories. Three of these—condition, nutrition, and health—can, in a broad sense be considered as components of physiology at an individual and population level as all contribute significantly to an animal’s physiological state. Our fourth subcategory includes studies that had adopted more ‘traditional’ physiological approaches.

- 1. *Condition*

To receive a ‘yes’ for this subcategory the study needed to have considered one or more of the following:

1. Degree of distress associated with the release process (reactions of animals assessed qualitatively by observation, with no endocrine monitoring); and
2. Body condition. Many studies scored condition using visual appraisals of physical appearance or as indices of body fat, but more-quantitative methods using residuals derived from regressions of body mass on body length or other linear measures were also included. Studies that considered body condition *post hoc* were excluded, as were those that used body condition to age the study subjects.
   1. *Nutrition*

To receive a ‘yes’ for this subcategory the study needed to have considered one or more of the following:

1. Pre-release diet; and
2. Post-release diet (if relevant, e.g. supplemental feeding).
   1. *Health*

To receive a ‘yes’ for this subcategory the study needed to have considered one or more of the following:

1. Health status of the source population;
2. Health check (generally by a veterinarian);
3. Vaccinations;
4. Parasite management;
5. Disease screening;
6. Quarantine; and
7. Presence of unfamiliar diseases/parasites at the release site.
   1. *‘Traditional’ physiology*

To receive a ‘yes’ for this subcategory the study needed to have considered one or more of the following:

1. Stress associated with the release process (with monitoring of glucocorticoids or other ‘stress hormones’);
2. Water and micronutrient balance;
3. Thermoregulation; and
4. Immunoecology.

*(d) Habitat*

To receive a ‘yes’ for this category the study needed to have considered one or more of the following:

1. Suitability of habitat for the target species at the new location; this included, for example, considerations of habitat fragmentation, human/animal activities in the area, whether the area was protected or multi-use, whether adequate food, water and shelter were available, and whether the reintroduction was specified as taking place in the species’ indigenous range; and
2. Whether specific enemy-shelter such as a predator-proof fence was available or necessary.

*(e) Key Threatening Processes (KTPs)*

Populations of many species that have been extirpated or severely compromised in an area can be impacted by one or more key threatening processes (KTPs). For example, a release of brush-tailed bettongs (*Bettongia penicillata*) in Australia was managed by the control of one threat, the predatory red fox (*Vulpes vulpes*), but was compromised by inattention to another threat – predation from the feral cat (*Felis catus*) ([Priddel & Wheeler, 2004](#_ENREF_99)). In this review we scored known or identified KTPs as present (P) or absent (A), but note that often there was minimal information on threatening processes. If some but not all known KTPs had been eliminated prior to a reintroduction, KTPs were considered to be present, but if all known KTPs had been eliminated they were considered absent.

**(3) Quantifying reintroduction success**

The most commonly used definition of ‘success’ in reintroduction programs is whether the programs result in self-sustaining populations of the target species (Griffith *et al*., 1989; Fischer & Lindenmayer, 2000). However, for several reasons this ostensibly simple definition can be difficult to meet.

Firstly, few studies specify how long a program should be monitored to confirm viability, although many acknowledge that years or decades may be required for success to become clear (Griffith *et al.,*1989, Fischer & Lindenmayer, 2000; Seddon *et al.,* 2007; Armstrong & Seddon, 2008). Post-release monitoring can necessarily continue only for a finite length of time in most projects; unless a target population becomes unambiguously self-sustaining in this time, success may be illusory and the true outcome of the project will remain unknown.

Secondly, as natural populations frequently decline to low numbers and become locally extinct, reintroduction success as measured by population persistence should also take account of the ‘background’ rate of population loss. For example, the ‘average’ species consists of 220 populations, of which about 8% are lost every decade ([Hughes, Daily & Ehrlich, 1997](#_ENREF_55)). From this we might expect a 92% chance for a reintroduced population to survive 10 years. Even this would be generous, however, given that most reintroduced populations are small and solitary and lack connectivity with other populations that could bolster them via dispersal or migration. Persistence times also are likely to be species-specific and perhaps less for already-threatened species than others. These considerations suggest that a 10-year background success rate for reintroduced populations could be set at 90% or less, but with considerable uncertainty due to the biology of the target species.

Thirdly, what appears to be a self-sustaining population at one point can decline rapidly, thereby reducing the chance of long-term persistence. For example, unexpected but catastrophic flooding greatly reduced the survival of reintroduced riparian brush rabbits (*Sylvilagus bachmani riparius*) in an otherwise successful program in California ([Hamilton *et al*., 2010](#_ENREF_48)). Even without such stochastic events, researchers may remain uncertain whether they have established a viable and self-sustaining population if they do not create measurable objectives against which to test a project’s performance ([Sheean *et al*., 2012](#_ENREF_118)); the lack of clarity about the achievement of success also makes subsequent meta-analyses very difficult ([Nakagawa & Cuthill, 2007](#_ENREF_89)).

To overcome these difficulties, we attempted to create specific criteria to determine the success of individual reintroduction projects in a repeatable and rigorous manner. As our review concerns reintroductions carried out over varying lengths of time, we considered each study on its own merits. In the first instance we evaluated success, or otherwise, of a reintroduction project based on each study’s self-evaluation. However, some studies, while considering their project a success, failed to meet their stated aims or, in our reading of the results, failed to state reasonable reasons for considering the project a success. Therefore, in addition to self-reported success and failure, we introduce a binary category for projects deemed successful, this being to denote ‘high’ or ‘low’ success.

1. High success was determined if:
   1. The reintroduction confirmed that a stable and/or increasing population was established during the study period; or
   2. The project achieved its specified aims. For example, a project evaluating the effects of pre-release experience of elk (*Cervus elaphus*) with wolves (*Canis lupus*) and human hunters showed that experienced animals survived longer post-release, which was the specified aim ([Frair *et al*., 2007](#_ENREF_38)); or
   3. The project initially showed poor results, but researchers improved them by altering protocols over time using information gleaned in earlier years (if releases took place over multiple years).
2. Low success was determined if:
   1. The study reported high success but failed to show conclusive results. For example, in a black bear (*Ursus americanus*) reintroduction that measured two different release techniques, >50% of study animals died or were unable to be included in the analyses due to lack of knowledge of their whereabouts ([Eastridge & Clark, 2001](#_ENREF_31));
   2. A threatening problem was present and could not be resolved, such as low genetic diversity due to small founder numbers or the presence of a key threatening process;
   3. Stochastic events occurred and significantly affected the project’s results. For example, during the Iraq war the flight of Bedouins from Kuwait and Iraq to Jordan led to a doubling of the livestock population in the host country. This led to overgrazing, reduced water supplies and higher prevalence of disease and parasites in Jordanian habitats, compromising the reintroduction of oryx (*Oryx leucoryx*) as a result ([Harding *et al*., 2007](#_ENREF_49));
   4. The sample size was too limited to have resulted in a self-sustaining population as, for example, in the reintroduction of a single orang-utan (*Pongo abelii*) to Sumatra ([Cocks & Bullo, 2008](#_ENREF_19));
   5. There was limited scope for population expansion and persistence. For example, despite the establishment of a reproducing population of lions (*Panthera leo*) in Phinda private game reserve, the population remained small and isolated, with little scope for connection to other isolated populations and for addressing the long-term conservation problems of the species ([Hunter *et al*., 2007](#_ENREF_56)); or
   6. The time of monitoring was too short to span even one breeding season. For example, a release of Pere David’s deer (*Elaphurus davidianus*) in China spanned less than six months of monitoring ([Hu & Jiang, 2002](#_ENREF_54)).

**Table S1: Length of post-release monitoring**

| **Length of post-release monitoring** | **Total Number** | **Failure** | **Success** |
| --- | --- | --- | --- |
| <1 month | 3 | 2 | 1 |
| 1-6 months | 15 | 1 | 14 |
| 6-12 months | 11 | 5 | 6 |
| 1-2 years | 23 | 3 | 20 |
| 2-5 years | 34 | 3 | 31 |
| 5-10 years | 20 | 2 | 18 |
| 10-20 years | 10 | 2 | 8 |
| 20+ years | 3 | 0 | 3 |
