# Supplementary material for: Physiology in conservation translocations
Source: Conserv Physiol. 2014 Dec 17;2(1):cou054. doi: 10.1093/conphys/cou054 (PMC4732500; doi:10.1093/conphys/cou054)
Supplement: Supplementary Data [file supp_cou054_cou054supp_file.docx]

**Supplementary material: References**

**Pre-release references**

Abbott, I. 2000. Improving the conservation of threatened and rare mammal species through translocation to islands: case study Western Australia. Biological Conservation **93**:195-201.

Angulo, F. and J. Barrio. 2004. Evaluation of a potential reintroduction site for the white-winged guan Penelope albipennis (Aves, Cracidae) in northern Peru. Oryx **38**:448-451.

Asa, C. S. 2010. The importance of reproductive management and monitoring in canid husbandry and endangered‐species recovery. International Zoo Yearbook **44**:102-108.

Bosé, M., F. Sarrazin, P. Le Gouar, C. Arthur, J. Lambourdière, J. P. Choisy, S. Henriquet, P. Lecuyer, M. Richard, and C. Tessier. 2007. Does sex matter in reintroduction of griffon vultures Gyps fulvus? Oryx **41**:503-508.

Bright, P. W. and T. J. Smithson. 2001. Biological invasions provide a framework for reintroductions: selecting areas in England for pine marten releases. Biodiversity and Conservation **10**:1247-1265.

Carroll, C., M. K. Phillips, N. H. Schumaker, and D. W. Smith. 2003. Impacts of Landscape Change on Wolf Restoration Success: Planning a Reintroduction Program Based on Static and Dynamic Spatial Models. Conservation Biology **17**:536-548.

Casimir, D. L., A. Moehrenschlager, and R. M. R. Barclay. 2007. Factors Influencing Reproduction in Captive Vancouver Island Marmots: Implications for Captive Breeding and Reintroduction Programs. Journal of Mammalogy **88**:1412-1419.

Cindy A, T., M. Frank T. Van, and C. Joseph D. 2009. Identifying Suitable Sites for Florida Panther Reintroduction. Journal of Wildlife Management **70**:752-763.

David, J. A. W., L. K. John, and W. W. L. Peter. 2009. Tree Squirrel Introduction: A Theoretical Approach with Population Viability Analysis. Journal of Mammalogy **88**:1271-1279.

Didier, K. A. and W. F. Porter. 1999. Large-Scale Assessment of Potential Habitat to Restore Elk to New York State. Wildlife Society Bulletin **27**:409-418.

Doug P, A., R. Elizabeth H, L. Rebecca M, and R. Don. 2009. Modeling Vital Rates of a Reintroduced New Zealand Robin Population as a Function of Predator Control. Journal of Wildlife Management **70**:1028-1036.

Elsbeth McPhee, M. 2004. Generations in captivity increases behavioral variance: considerations for captive breeding and reintroduction programs. Biological Conservation **115**:71-77.

Emily K, L., A. Roger D, and J. R. Olin E. Rhodes. 2009. Genetic Composition of Wild Turkeys in Kansas Following Decades of Translocations. Journal of Wildlife Management **70**:1698-1703.

Etterson, M. A. 2003. Conspecific attraction in loggerhead shrikes: implications for habitat conservation and reintroduction. Biological Conservation **114**:199-205.

Graham, T. U. 1999. Method for attaching radio transmitters to medium-sized reptiles: Trials on tuatara (Sphenodon punctatus). Herpetological Review **30**:151.

Gross, J. E., F. J. Singer, and M. E. Moses. 2000. Effects of Disease, Dispersal, and Area on Bighorn Sheep Restoration. Restoration Ecology **8**:25-37.

Gusset, M., O. Jakoby, M. S. Müller, M. J. Somers, R. Slotow, and V. Grimm. 2009. Dogs on the catwalk: Modelling re-introduction and translocation of endangered wild dogs in South Africa. Biological Conservation **142**:2774-2781.

Hartup, B. K., G. H. Olsen, and N. M. Czekala. 2005. Fecal corticoid monitoring in whooping cranes (Grus americana) undergoing reintroduction. Zoo Biology **24**:15-28.

Heaton, J. S., L. Benvenuti, K. E. Nussear, T. C. Esque, R. D. Inman, F. M. Davenport, T. E. Leuteritz, P. A. Medica, N. W. Strout, and P. A. Burgess. 2008. Spatially explicit decision support for selecting translocation areas for Mojave desert tortoises. Biodiversity and Conservation **17**:575-590.

Hetherington, D. A. and M. L. Gorman. 2007. Using prey densities to estimate the potential size of reintroduced populations of Eurasian lynx. Biological Conservation **137**:37-44.

Johnsingh, A. J. T., S. P. Goyal, and Q. Qureshi. 2007. Preparations for the reintroduction of Asiatic lion Panthera leo persica into Kuno Wildlife Sanctuary, Madhya Pradesh, India. Oryx **41**:93-96.

Kramer-Schadt, S., E. Revilla, and T. Wiegand. 2005. Lynx reintroductions in fragmented landscapes of Germany: Projects with a future or misunderstood wildlife conservation? Biological Conservation **125**:169-182.

Martínez-Meyer, E., A. T. Peterson, J. I. Servín, and L. F. Kiff. 2006. Ecological niche modelling and prioritizing areas for species reintroductions. Oryx **40**:411-418.

Mathews, F., M. Orros, G. McLaren, M. Gelling, and R. Foster. 2005. Keeping fit on the ark: assessing the suitability of captive-bred animals for release. Biological Conservation **121**:569-577.

McPhee, M. E. and E. D. Silverman. 2004. Increased Behavioral Variation and the Calculation of Release Numbers for Reintroduction Programs. Conservation Biology **18**:705-715.

Merrill, T., D. J. Mattson, R. G. Wright, and H. B. Quigley. 1999. Defining landscapes suitable for restoration of grizzly bears Ursus arctos in Idaho. Biological Conservation **87**:231-248.

Metzger, K. L., A. R. E. Sinclair, K. L. I. Campbell, R. Hilborn, J. G. C. Hopcraft, S. A. R. Mduma, and R. M. Reich. 2007. Using historical data to establish baselines for conservation: The black rhinoceros ( Diceros bicornis) of the Serengeti as a case study. Biological Conservation **139**:358-374.

Negro, J. J. and M. a. J. Torres. 1999. Genetic variability and differentiation of two bearded vulture Gypaetus barbatus populations and implications for reintroduction projects. Biological Conservation **87**:249-254.

Niemuth, N. D. 2003. Identifying Landscapes for Greater Prairie Chicken Translocation Using Habitat Models and GIS: A Case Study. Wildlife Society Bulletin **31**:145-155.

O’Toole, L., A. H. Fielding, and P. F. Haworth. 2002. Re-introduction of the golden eagle into the Republic of Ireland. Biological Conservation **103**:303-312.

Olech, W. and K. Perzanowski. 2002. A genetic background for reintroduction program of the European bison ( Bison bonasus) in the Carpathians. Biological Conservation **108**:221-228.

Osborne, P. E. 2005. Key issues in assessing the feasibility of reintroducing the great bustard Otis tarda to Britain. Oryx **39**:22-29.

Pedrono, M., L. L. Smith, J. Clobert, M. Massot, and F. Sarrazin. 2004. Wild-captive metapopulation viability analysis. Biological Conservation **119**:463-473.

Pertoldi, C., J. J. Negro, J. Muñoz, F. Barbanera, and H. Garrido. 2006. Introduction or reintroduction? Last resorts for the latest bird to become extinct in Europe, the Andalusian hemipode Turnix sylvatica sylvatica. Biodiversity and Conservation **15**:3895-3908.

Roe, C. M. and K. A. Roe. 2003. Habitat Selection Guidelines for Black-Tailed Prairie Dog Relocations. Wildlife Society Bulletin **31**:1246-1253.

Saenz, D., K. A. Baum, R. N. Conner, D. C. Rudolph, and R. Costa. 2002. Large-Scale Translocation Strategies for Reintroducing Red-Cockaded Woodpeckers. The Journal of Wildlife Management **66**:212-221.

South, A., S. Rushton, and D. Macdonald. 2000. Simulating the proposed reintroduction of the European beaver ( Castor fiber) to Scotland. Biological Conservation **93**:103-116.

Steury, T. D. and D. L. Murray. 2004. Modeling the reintroduction of lynx to the southern portion of its range. Biological Conservation **117**:127-141.

Tash, J. P. and J. A. Litvaitis. 2007. Characteristics of occupied habitats and identification of sites for restoration and translocation of New England cottontail populations. Biological Conservation **137**:584-598.

Tenhumberg, B., A. J. Tyre, K. Shea, and H. P. Possingham. 2004. Linking Wild and Captive Populations to Maximize Species Persistence: Optimal Translocation Strategies. Conservation Biology **18**:1304-1314.

van Heezik, Y., P. Lei, R. Maloney, and E. Sancha. 2005. Captive breeding for reintroduction: influence of management practices and biological factors on survival of captive kaki (black stilt). Zoo Biology **24**:459-474.

Van Manen, F. T., J. D. Clark, and M. E. Cartwright. 2007. Identifying Sites for Elk Restoration in Arkansas. The Journal of Wildlife Management **71**:1393-1403.

Wakamiya, S. M. and C. L. Roy. 2009. Use of monitoring data and population viability analysis to inform reintroduction decisions: Peregrine falcons in the Midwestern United States. Biological Conservation **142**:1767-1776.

Yan, P., X. Wu, Y. Wang, Z. Jiang, C. Gu, and C. Wang. 2006. AFLP Analysis of genetic variation on captive‐bred chinese alligators: an application to select individuals for release. Zoo Biology **25**:479-490.

**Conservation translocation references**

Aaltonen, K., A. A. Bryant, J. A. Hostetler, and M. K. Oli. 2009. Reintroducing endangered Vancouver Island marmots: Survival and cause-specific mortality rates of captive-born versus wild-born individuals. Biological Conservation **142**:2181-2190.

Alan, P. and L. Colin. 2009. The translocation and post release settlement of red squirrels Sciurus vulgaris to a previously uninhabited woodland. Biodiversity and Conservation **18**:3205-3218.

Allison, C. A. 2002. Ten year of conservation research on Cuban rock iguanas. Herpetological Review **33**:119.

Ancrenaz, M., J. Paredes, M. Vacher-Vallas, C. Vidal, B. Goossens, M. W. Bruford, and A. Jamart. 2001. Conservation Biology Framework for the Release of Wild-Born Orphaned Chimpanzees into the Conkouati Reserve, Congo. Conservation Biology **15**:1247-1257.

Armstrong, D. P., I. Castro, J. C. Alley, B. Feenstra, and J. K. Perrott. 1999. Mortality and behaviour of hihi, an endangered New Zealand honeyeater, in the establishment phase following translocation. Biological Conservation **89**:329-339.

Armstrong, D. P. and J. G. Ewen. 2002. Dynamics and Viability of a New Zealand Robin Population Reintroduced to Regenerating Fragmented Habitat. Conservation Biology **16**:1074-1085.

Banks, P. B., K. Norrdahl, and E. Korpimäki. 2002. Mobility decisions and the predation risks of reintroduction. Biological Conservation **103**:133-138.

Bar-David, S., D. Saltz, T. Dayan, A. Perelberg, and A. Dolev. 2005. Demographic Models and Reality in Reintroductions: Persian Fallow Deer in Israel. Conservation Biology **19**:131-138.

Baxter, R. J., J. T. Flinders, and D. L. Mitchell. 2008. Survival, Movements, and Reproduction of Translocated Greater Sage-Grouse in Strawberry Valley, Utah. The Journal of Wildlife Management **72**:179-186.

Baxter, R. J., J. T. Flinders, D. G. Whiting, and D. L. Mitchell. 2009. Factors affecting nest-site selection and nest success of translocated greater sage grouse. Wildlife Research **36**:479-487.

Bellis, L. M., M. B. Martella, and J. L. Navarro. 2004. Habitat use by wild and captive-reared greater rheas Rhea americana in agricultural landscapes in Argentina. Oryx **38**:304-310.

Benson, J. F. and M. J. Chamberlain. 2007. Space Use, Survival, Movements, and Reproduction of Reintroduced Louisiana Black Bears. The Journal of Wildlife Management [H.W. Wilson - GS] **71**:2393.

Biggins, D. E., A. Vargas, J. L. Godbey, and S. H. Anderson. 1999. Influence of prerelease experience on reintroduced black-footed ferrets ( Mustela nigripes). Biological Conservation **89**:121-129.

Brightsmith, D., J. Hilburn, A. del Campo, J. Boyd, R. Frisius, M. Frisius, D. Janik, and F. Guillen. 2005. The use of hand-raised psittacines for reintroduction: a case study of scarlet macaws ( Ara macao) in Peru and Costa Rica. Biological Conservation **121**:465-472.

Britt, A., C. Welch, and A. Katz. 2004a. Can small, isolated primate populations be effectively reinforced through the release of individuals from a captive population? Biological Conservation **115**:319-327.

Britt, A., C. Welch, A. Katz, B. Iambana, I. Porton, R. Junge, G. Crawford, C. Williams, and D. Haring. 2004b. The re-stocking of captive-bred ruffed lemurs (Varecia variegata variegata) into the Betampona Reserve, Madagascar: methodology and recommendations. Biodiversity and Conservation **13**:635-657.

Buner, F., M. Jenny, N. Zbinden, and B. Naef-Daenzer. 2005. Ecologically enhanced areas – a key habitat structure for re-introduced grey partridges Perdix perdix. Biological Conservation **124**:373-381.

Calvete, C., E. Angulo, R. Estrada, S. Moreno, and R. Villafuerte. 2005. Quarantine Length and Survival of Translocated European Wild Rabbits. The Journal of Wildlife Management **69**:1063-1072.

Calvete, C. and R. Estrada. 2004. Short-term survival and dispersal of translocated European wild rabbits. Improving the release protocol. Biological Conservation **120**:507-516.

Cheyne, S. M., D. J. Chivers, and J. Sugardjito. 2008. Biology and behaviour of reintroduced gibbons. Biodiversity and Conservation **17**:1741-1751.

Chiarello, A. G., D. J. Chivers, C. Bassi, M. A. F. Maciel, L. S. Moreira, and M. Bazzalo. 2004. A translocation experiment for the conservation of maned sloths, Bradypus torquatus (Xenarthra, Bradypodidae). Biological Conservation **118**:421-430.

Chrysogaster, N., I. Smales, P. Brown, P. Menkhorst, M. Holdsworth, and P. Holz. 2000. Contribution of captive management of Orange‐bellied parrots to the recovery programme for the species in Australia. International Zoo Yearbook **37**:171-178.

Cocks, L. and K. Bullo. 2008. The processes for releasing a zoo‐bred Sumatran orang‐utan Pongo abelii at Bukit Tigapuluh National Park, Jambi, Sumatra. International Zoo Yearbook **42**:183-189.

Cristinacce, A., A. Ladkoo, R. Switzer, L. Jordan, V. Vencatasamy, F. de Ravel Koenig, C. Jones, and D. Bell. 2008. Captive breeding and rearing of critically endangered Mauritius fodies Foudia rubra for reintroduction. Zoo Biology **27**:255-268.

Diefenbach, D. R., L. A. Hansen, R. J. Warren, and M. J. Conroy. 2006. Spatial Organization of a Reintroduced Population of Bobcats. Journal of Mammalogy **87**:394-401.

Dolev, A., D. Saltz, S. Bar-David, and Y. Yom-Tov. 2002. Impact of Repeated Releases on Space-Use Patterns of Persian Fallow Deer. The Journal of Wildlife Management **66**:737-746.

Dzialak, M. R., M. J. Lacki, K. M. Carter, K. Huie, and J. J. Cox. 2006. An Assessment of Raptor Hacking during a Reintroduction. Wildlife Society Bulletin **34**:542-547.

Eastridge, R. and J. D. Clark. 2001. Evaluation of 2 Soft-Release Techniques to Reintroduce Black Bears. Wildlife Society Bulletin **29**:1163-1174.

Engelhardt, K. A. M., J. A. Kadlec, V. L. Roy, and J. A. Powell. 2000. Evaluation of translocation criteria: case study with trumpeter swans ( Cygnus buccinator). Biological Conservation **94**:173-181.

Faulhaber, C. A., N. J. Silvy, R. R. Lopez, P. A. Frank, and M. J. Peterson. 2006. Reintroduction of Lower Keys Marsh Rabbits. Wildlife Society Bulletin **34**:1198-1202.

Field, K. J., C. R. Tracy, P. A. Medica, R. W. Marlow, and P. S. Corn. 2007. Return to the wild: Translocation as a tool in conservation of the Desert Tortoise ( Gopherus agassizii). Biological Conservation **136**:232-245.

Foresman, K. R. and M. R. Matchett. 2005. Efficacy of Translocations for Restoring Populations of Black-Tailed Prairie Dogs. Wildlife Society Bulletin **33**:842-850.

Frair, J. L., E. H. Merrill, and J. R. Allen. 2007. Know Thy Enemy: Experience Affects Elk Translocation Success in Risky Landscapes. The Journal of Wildlife Management [H.W. Wilson - GS] **71**:541.

Goldsworthy, S. D., M. Giese, R. P. Gales, N. Brothers, and J. Hamill. 2000. Effects of the Iron Baron oil spill on little penguins (Eudyptula minor). II. Post-release survival of rehabilitated oiled birds. Wildlife Research **27**:573.

Goossens, B., J. M. Setchell, E. Tchidongo, E. Dilambaka, C. Vidal, M. Ancrenaz, and A. Jamart. 2005. Survival, interactions with conspecifics and reproduction in 37 chimpanzees released into the wild. Biological Conservation **123**:461-475.

Green, A. J., C. Fuentes, J. Figuerola, C. Viedma, and N. Ramón. 2005. Survival of Marbled Teal ( Marmaronetta angustirostris) released back into the wild. Biological Conservation **121**:595-601.

Groombridge, J. J., J. G. Massey, J. C. Bruch, T. Malcolm, C. N. Brosius, M. M. Okada, B. Sparklin, J. S. Fretz, and E. A. VanderWerf. 2004. An attempt to recover the Po'ouli by translocation and an appraisal of recovery strategy for bird species of extreme rarity. Biological Conservation **118**:365-375.

Hamilton, L. P., P. A. Kelly, D. F. Williams, D. A. Kelt, and H. U. Wittmer. 2010. Factors associated with survival of reintroduced riparian brush rabbits in California. Biological Conservation **143**:999-1007.

Harding, L. E., O. F. Abu-Eid, N. Hamidan, and A. al Sha'lan. 2007. Reintroduction of the Arabian oryx Oryx leucoryx in Jordan: war and redemption. Oryx **41**:478-487.

Hardman, B. and D. Moro. 2006a. Importance of diurnal refugia to a hare-wallaby reintroduction in Western Australia. Wildlife Research **33**:355-359.

Hardman, B. and D. Moro. 2006b. Optimising reintroduction success by delayed dispersal: Is the release protocol important for hare-wallabies? Biological Conservation **128**:403-411.

Hu, H. and Z. Jiang. 2002. Trial release of Père David's deer Elaphurus davidianus in the Dafeng Reserve, China. Oryx **36**:196-199.

Hunter, L. T. B., K. Pretorius, L. C. Carlisle, M. Rickelton, C. Walker, R. Slotow, and J. D. Skinner. 2007. Restoring lions Panthera leo to northern KwaZulu-Natal, South Africa: short-term biological and technical success but equivocal long-term conservation. Oryx **41**:196-204.

Israel, D. P., E. W. Dominique, R. L. Roel, J. S. Nova, S. D. Donald, A. M. Robert, and A. F. Philip. 2009. Evaluation of the Efficacy of Florida Key Deer Translocations. Journal of Wildlife Management **72**:1069-1075.

Jeffery, R. B., A. B. Christine, and J. B. Ronald. 2009. Effectiveness of Short-Distance Translocation and its Effects on Western Rattlesnakes. Journal of Wildlife Management **73**:419-425.

Jenny, J. P., W. Heinrich, A. B. Montoya, B. Mutch, C. Sandfort, and W. G. Hunt. 2004. Progress in Restoring the Aplomado Falcon to Southern Texas. Wildlife Society Bulletin **32**:276-285.

Jiang, Z., C. Yu, Z. Feng, L. Zhang, J. Xia, Y. Ding, and N. Lindsay. 2000. Reintroduction and Recovery of Père David's Deer in China. Wildlife Society Bulletin **28**:681-687.

John, D. L., L. S. Gary, and S. Skip. 2009. Demography of Reintroduced Eastern Bluebirds and Brown-Headed Nuthatches. Journal of Wildlife Management **73**:955-964.

Johnson, S. A. and K. A. Berkley. 1999. Restoring River Otters in Indiana. Wildlife Society Bulletin **27**:419-427.

Joy, M. H., J. P. Steven, and E. D. Michael. 2009. Effects of Relocation on Movements and Home Ranges of Eastern Box Turtles. Journal of Wildlife Management **72**:772-777.

King, S. R. B. and J. Gurnell. 2005. Habitat use and spatial dynamics of takhi introduced to Hustai National Park, Mongolia. Biological Conservation **124**:277-290.

Kreger, M. D., J. S. Hatfield, I. Estevez, G. F. Gee, and D. A. Clugston. 2006. Behavioral profiles of the captive juvenile whooping crane as an indicator of post‐release survival. Zoo Biology **25**:11-24.

Kuehler, C., P. Harrity, E. Tweed, S. Fancy, B. Woodworth, T. Telfer, A. Lieberman, P. Oesterle, T. Powers, M. Kuhn, J. Kuhn, J. Nelson, T. Snetsinger, and C. Herrmann. 2000. Development of restoration techniques for Hawaiian thrushes: Collection of wild eggs, artificial incubation, hand‐rearing, captive‐breeding, and re‐introduction to the wild. Zoo Biology **19**:263-277.

Lander, M. E. 2003. Rehabilitation and Post-Release Monitoring of Steller Sea Lion Pups Raised in Captivity. Wildlife Society Bulletin **31**:1047-1053.

Lapidge, S. J. 2005. Reintroduction increased vitamin E and condition in captive-bred yellow-footed rock wallabies Petrogale xanthopus. Oryx **39**:56-64.

Larkin, J. L., J. J. Cox, M. W. Wichrowski, M. R. Dzialak, and D. S. Maehr. 2004. Influences on Release‐Site Fidelity of Translocated Elk. Restoration Ecology **12**:97-105.

Larkin, J. L., D. S. Maehr, J. J. Cox, D. C. Bolin, and M. W. Wichrowski. 2003. Demographic Characteristics of a Reintroduced Elk Population in Kentucky. The Journal of Wildlife Management **67**:467-476.

Leech, T. J., E. Craig, B. Beaven, D. K. Mitchell, and P. J. Seddon. 2007. Reintroduction of rifleman Acanthisitta chloris to Ulva Island, New Zealand: evaluation of techniques and population persistence. Oryx **41**:369-375.

Luiz Pereira, S. and A. Wajntal. 1999. Reintroduction of guans of the genus Penelope (Cracidae, Aves) in reforested areas in Brazil: assessment by DNA fingerprinting. Biological Conservation **87**:31-38.

Manire, C. A., C. J. Walsh, H. L. Rhinehart, D. E. Colbert, D. R. Noyes, and C. A. Luer. 2003. Alterations in blood and urine parameters in two florida manatees (Trichechus manatus latirostris) from simulated conditions of release following rehabilitation. Zoo Biology **22**:103-120.

Maran, T., M. Põdra, M. Põlma, and D. W. Macdonald. 2009. The survival of captive-born animals in restoration programmes – Case study of the endangered European mink Mustela lutreola. Biological Conservation **142**:1685-1692.

Mathews, F., D. Moro, R. Strachan, M. Gelling, and N. Buller. 2006. Health surveillance in wildlife reintroductions. Biological Conservation **131**:338-347.

Miskelly, C. M., G. A. Taylor, H. Gummer, and R. Williams. 2009. Translocations of eight species of burrow-nesting seabirds (genera Pterodroma, Pelecanoides, Pachyptila and Puffinus: Family Procellariidae). Biological Conservation **142**:1965-1980.

Mitchell A, L., G. Clifton P, M. Michael E, R. Charles J, and S. Nova J. 2009. Survival, Movements, and Reproduction of Released Captive-reared Attwater's Praire-Chicken. Journal of Wildlife Management **69**:1251-1258.

Molony, S. E., C. V. Dowding, P. J. Baker, I. C. Cuthill, and S. Harris. 2006. The effect of translocation and temporary captivity on wildlife rehabilitation success: An experimental study using European hedgehogs ( Erinaceus europaeus). Biological Conservation **130**:530-537.

Moorhouse, T. P., M. Gelling, and D. W. Macdonald. 2009. Effects of habitat quality upon reintroduction success in water voles: Evidence from a replicated experiment. Biological Conservation **142**:53-60.

Moro, D. 2003. Translocation of captive-bred dibblers Parantechinus apicalis (Marsupialia: Dasyuridae) to Escape Island, Western Australia. Biological Conservation **111**:305-315.

Moseby, K. E. and E. O. Donnell. 2003. Reintroduction of the greater bilby, (Reid) (Marsupialia : Thylacomyidae), to northern South Australia: survival, ecology and notes on reintroduction protocols. Wildlife Research **30**:15-27.

Mumme, R. L. and T. H. Below. 1999. Evaluation of Translocation for the Threatened Florida Scrub-Jay. The Journal of Wildlife Management **63**:833-842.

Munkwitz, N. M., J. M. Turner, E. L. Kershner, S. M. Farabaugh, and S. R. Heath. 2005. Predicting release success of captive‐reared loggerhead shrikes (Lanius ludovicianus) using pre‐release behavior. Zoo Biology **24**:447-458.

Murrow, J. L., J. D. Clark, and E. K. Delozier. 2009. Demographics of an Experimentally Released Population of Elk in Great Smoky Mountains National Park. The Journal of Wildlife Management **73**:1261-1268.

Nelson, N. J., S. N. Keall, D. Brown, and C. H. Daugherty. 2002. Establishing a New Wild Population of Tuatara (Sphenodon guntheri). Conservation Biology **16**:887-894.

Nichols, R. K., J. Steiner, L. G. Woolaver, E. Williams, A. A. Chabot, and K. Tuininga. 2010. Conservation initiatives for an endangered migratory passerine: field propagation and release. Oryx **44**:171-177.

Ostermann, S. D., J. R. Deforge, and W. D. Edge. 2001. Captive Breeding and Reintroduction Evaluation Criteria: A Case Study of Peninsular Bighorn Sheep. Conservation Biology **15**:749-760.

Ostro, L. E. T., S. C. Silver, F. W. Koontz, T. P. Young, and R. H. Horwich. 1999. Ranging behavior of translocated and established groups of black howler monkeys Alouatta pigra in Belize, Central America. Biological Conservation **87**:181-190.

Pedrono, M. and A. Sarovy. 2000. Trial release of the world's rarest tortoise Geochelone yniphora in Madagascar. Biological Conservation **95**:333-342.

Peignot, P., M. J. E. Charpentier, N. Bout, O. Bourry, U. Massima, O. Dosimont, R. Terramorsi, and E. J. Wickings. 2008. Learning from the first release project of captive-bred mandrills Mandrillus sphinx in Gabon. Oryx **42**:122-131.

Pereladova, O. B., A. J. Sempéeré, N. V. Soldatova, V. U. Dutov, G. Fisenko, and V. E. Flint. 1999. Przewalski's horse—adaptation to semi-wild life in desert conditions. Oryx **33**:47-58.

Pierre, J. P. 1999. Reintroduction of the South Island saddleback ( Philesturnus carunculatus carunculatus): dispersal, social organisation and survival. Biological Conservation **89**:153-159.

Pinter-Wollman, N., L. A. Isbell, and L. A. Hart. 2009. Assessing translocation outcome: Comparing behavioral and physiological aspects of translocated and resident African elephants ( Loxodonta africana). Biological Conservation **142**:1116-1124.

Pople, A. R., J. Lowry, G. Lundie-Jenkins, T. F. Clancy, H. I. McCallum, D. Sigg, D. Hoolihan, and S. Hamilton. 2001. Demography of bridled nailtail wallabies translocated to the edge of their former range from captive and wild stock. Biological Conservation **102**:285-299.

Priddel, D., N. Carlile, and R. Wheeler. 2006. Establishment of a new breeding colony of Gould’s petrel ( Pterodroma leucoptera leucoptera) through the creation of artificial nesting habitat and the translocation of nestlings. Biological Conservation **128**:553-563.

Priddel D. and Wheeler R. 2004. An experimental translocation of brush-tailed bettongs (Bettongia penicillata) to western New South Wales. Wildlife Research **31**:421-432.

Richard-Hansen, C., J. C. Vié, and B. t. de Thoisy. 2000. Translocation of red howler monkeys ( Alouatta seniculus) in French Guiana. Biological Conservation **93**:247-253.

Richard, N. C., D. C. Rudolph, S. Daniel, R. S. Richard, and J. B. Shirley. 2003. Growth Rates and Post-Release Survival of Captive Neonate Timber Rattlesnakes, Crotalus horridus. Page 314. Society for the Study of Amphibians and Reptiles, St. Louis.

Richards, J. D. and J. Short. 2003. Reintroduction and establishment of the western barred bandicoot Perameles bougainville (Marsupialia: Peramelidae) at Shark Bay, Western Australia. Biological Conservation **109**:181-195.

Rittenhouse, C. D., J. J. Millspaugh, M. W. Hubbard, S. L. Sheriff, and W. D. Dijak. 2008. Resource Selection by Translocated Three-Toed Box Turtles in Missouri. The Journal of Wildlife Management **72**:268-275.

Roche, E. A., F. J. Cuthbert, and T. W. Arnold. 2008. Relative fitness of wild and captive-reared piping plovers: Does egg salvage contribute to recovery of the endangered Great Lakes population? Biological Conservation **141**:3079-3088.

Rosatte, R., J. Hamr, J. Young, I. Filion, and H. Smith. 2007. The Restoration of Elk (Cervus elaphus) in Ontario, Canada: 1998–2005. Restoration Ecology **15**:34-43.

Rouco, C., P. Ferreras, F. Castro, and R. Villafuerte. 2008. The effect of exclusion of terrestrial predators on short-term survival of translocated European wild rabbits. Wildlife Research **35**:625-632.

Ryckman, M. J., R. C. Rosatte, T. McIntosh, J. Hamr, and D. Jenkins. 2010. Postrelease Dispersal of Reintroduced Elk (Cervus elaphus) in Ontario, Canada. Restoration Ecology **18**:173-180.

Saltz, D., M. Rowen, and D. I. Rubenstein. 2000. The Effect of Space-Use Patterns of Reintroduced Asiatic Wild Ass on Effective Population Size. Conservation Biology **14**:1852-1861.

Santos, T., J. Pérez-Tris, R. Carbonell, J. L. Tellería, and J. A. Díaz. 2009. Monitoring the performance of wild-born and introduced lizards in a fragmented landscape: Implications for ex situ conservation programmes. Biological Conservation **142**:2923-2930.

Shier, D. M. 2006. Effect of Family Support on the Success of Translocated Black-Tailed Prairie Dogs. Conservation Biology **20**:1780-1790.

Shier, D. M. and D. H. Owings. 2006. Effects of predator training on behavior and post-release survival of captive prairie dogs ( Cynomys ludovicianus). Biological Conservation **132**:126-135.

Short, J. and B. Turner. 2000. Reintroduction of the burrowing bettong Bettongia lesueur (Marsupialia: Potoroidae) to mainland Australia. Biological Conservation **96**:185-196.

Singer, F. J., V. C. Bleich, and M. A. Gudorf. 2000. Restoration of Bighorn Sheep Metapopulations in and Near Western National Parks. Restoration Ecology **8**:14-24.

Smeeton, C. and K. Weagle. 2000. The reintroduction of the swift fox Vulpes velox to South Central Saskatchewan, Canada. Oryx **34**:171-179.

Southgate, R. I., P. Christie, and K. Bellchambers. 2000. Breeding biology of captive, reintroduced and wild greater bilbies, Macrotis lagotis (Marsupialia : Peramelidae). Wildlife Research **27**:621.

Spalton, J. A., M. W. Lawerence, and S. A. Brend. 1999. Arabian oryx reintroduction in Oman: successes and setbacks. Oryx **33**:168-175.

Steven, J. L. 2000. Dietary adaptation of reintroduced yellow-footed rock-wallabies, (Marsupialia : Macropodidae), in the northern Flinders Ranges, South Australia. Wildlife Research **27**:195-201.

Stiver, S. J. and D. J. Delehanty. 2006. Using Sharp-Tailed Grouse Movement Patterns to Guide Release-Site Selection. Wildlife Society Bulletin **34**:1376-1382.

Støen, O.-G., M. L. Pitlagano, and S. R. Moe. 2009. Same-site multiple releases of translocated white rhinoceroses Ceratotherium simum may increase the risk of unwanted dispersal. Oryx **43**:580-585.

Stuart, A. and A. Kyle. 2001. The effect of familiarity and mound condition in translocations of the western pebble-mound mouse, , in the Pilbara region of Western Australia. Wildlife Research **28**:135-140.

Tavecchia, G., C. Viedma, A. Martínez-Abraín, M.-A. Bartolomé, J. A. Gómez, and D. Oro. 2009. Maximizing re-introduction success: Assessing the immediate cost of release in a threatened waterfowl. Biological Conservation **142**:3005-3012.

Tear, T. H. and E. D. Ables. 1999. Social system development and variability in a reintroduced Arabian oryx population. Biological Conservation **89**:199-207.

Theron M, T., S. D. Clay, and S. H. Lee. 2009. The Efficacy of Relocating Wild Northern Bobwhites Prior to Breeding Season. Journal of Wildlife Management **70**:914-921.

Towns, D. R. and S. M. Ferreira. 2001. Conservation of New Zealand lizards (Lacertilia: Scincidae) by translocation of small populations. Biological Conservation **98**:211-222.

Trevor, A. K. and J. N. Nancy. 2009. Ecology and Translocation-Aided Recovery of an Endangered Badger Population. Journal of Wildlife Management **72**:113-122.

Tuberville, T. D., T. M. Norton, B. D. Todd, and J. S. Spratt. 2008. Long-term apparent survival of translocated gopher tortoises: A comparison of newly released and previously established animals. Biological Conservation **141**:2690-2697.

Tweed, E. J., J. Kellerman, T. Telfer, J. T. Foster, B. L. Woodworth, P. Oesterle, C. Kuehler, A. A. Lieberman, A. T. Powers, K. Whitaker, and W. B. Monahan. 2003. Survival, dispersal, and home-range establishment of reintroduced captive-bred puaiohi, Myadestes palmeri. Biological Conservation **111**:1-9.

van Heezik, Y., R. F. Maloney, and P. J. Seddon. 2009. Movements of translocated captive-bred and released Critically Endangered kaki (black stilts) Himantopus novaezelandiae and the value of long-term post-release monitoring. Oryx **43**:639-647.

van Manen, F. T., B. A. Crawford, and J. D. Clark. 2000. Predicting Red Wolf Release Success in the Southeastern United States. The Journal of Wildlife Management **64**:895-902.

Van Zant, J. L. and M. C. Wooten. 2003. Translocation of Choctawhatchee beach mice ( Peromyscus polionotus allophrys): hard lessons learned. Biological Conservation **112**:405-413.

Vandel, J.-M., P. Stahl, V. Herrenschmidt, and E. Marboutin. 2006. Reintroduction of the lynx into the Vosges mountain massif: From animal survival and movements to population development. Biological Conservation **131**:370-385.

Wallace, M. T. and R. Buchholz. 2001. Translocation of Red-Cockaded Woodpeckers by Reciprocal Fostering of Nestlings. The Journal of Wildlife Management **65**:327-333.

Wanless, R. M., J. Wanless, J. Cunningham, P. A. R. Hockey, R. W. White, and R. Wiseman. 2002. The success of a soft-release reintroduction of the flightless Aldabra rail ( Dryolimnas [cuvieri] aldabranus) on Aldabra Atoll, Seychelles. Biological Conservation **107**:203-210.

Wayne, L. L. and R. S. Ronald. 2009. Reserve Size, Conspecific Density, and Translocation Success for Black Rhinoceros. Journal of Wildlife Management **72**:1059-1068.

Wear, B. J., R. Eastridge, and J. D. Clark. 2005. Factors Affecting Settling, Survival, and Viability of Black Bears Reintroduced to Felsenthal National Wildlife Refuge, Arkansas. Wildlife Society Bulletin **33**:1363-1374.

Zidon, R., D. Saltz, L. S. Shore, and U. Motro. 2009. Behavioral Changes, Stress, and Survival following Reintroduction of Persian Fallow Deer from Two Breeding Facilities. Conservation Biology **23**:1026-1035.

**Post release references**

Anderson, P., M. G. Turner, J. D. Forester, J. Zhu, M. S. Boyce, H. Beyer, and L. Stowell. 2005. Scale-Dependent Summer Resource Selection by Reintroduced Elk in Wisconsin, USA. The Journal of Wildlife Management **69**:298-310.

Armstrong, D. P. and J. G. Ewen. 2001. Assessing the value of follow-up translocations. Biological Conservation **101**:239-247.

Armstrong, D. P. and J. K. Perrott. 2000. An Experiment Testing Whether Condition and Survival are Limited by Food Supply in a Reintroduced Hihi Population. Conservation Biology **14**:1171-1181.

Aubry, K. B. and J. C. Lewis. 2003. Extirpation and reintroduction of fishers ( Martes pennanti) in Oregon: implications for their conservation in the Pacific states. Biological Conservation **114**:79-90.

Ausband, D. and A. Moehrenschlager. 2009. Long-range juvenile dispersal and its implication for conservation of reintroduced swift fox Vulpes velox populations in the USA and Canada. Oryx **43**:73-77.

Ausband, D. E. and K. R. Foresman. 2007. Swift fox reintroductions on the Blackfeet Indian Reservation, Montana, USA. Biological Conservation **136**:423-430.

Bodkin, J. L., B. E. Ballachey, M. A. Cronin, and K. T. Scribner. 1999. Population Demographics and Genetic Diversity in Remnant and Translocated Populations of Sea Otters. Conservation Biology **13**:1378-1385.

Bradley, J. S., L. R. Peters, and J. K. Christopher. 2009. Demographic and Genetic Evaluation of an American Marten Reintroduction. Journal of Mammalogy **87**:272-280.

Brown, J. L., M. W. Collopy, E. J. Gott, P. W. Juergens, A. B. Montoya, and W. G. Hunt. 2006. Wild-reared aplomado falcons survive and recruit at higher rates than hacked falcons in a common environment. Biological Conservation **131**:453-458.

Brown, S. K., J. M. Hull, D. R. Updike, S. R. Fain, and H. B. Ernest. 2009. Black Bear Population Genetics in California: Signatures of Population Structure, Competitive Release, and Historical Translocation. Journal of Mammalogy **90**:1066-1074.

Caroline van, D., R. Erica, E. W. d. V. Anneleen, F. d. B. Willem, J. H. G. H. René, and U. Dorj. 2009. Wolf Predation Among Reintroduced Przewalski Horses in Hustai National Park, Mongolia. Journal of Wildlife Management **73**:836-843.

Castro, I., D. H. Brunton, K. M. Mason, B. Ebert, and R. Griffiths. 2003. Life history traits and food supplementation affect productivity in a translocated population of the endangered Hihi (Stitchbird, Notiomystis cincta). Biological Conservation **114**:271-280.

Cotilla, I. and R. Villafuerte. 2007. Rabbit conservation: models to evaluate the effects of timing of restocking on recipient and donor populations. Wildlife Research **34**:247-252.

Davidson, A. D., R. R. Parmenter, and J. R. Gosz. 1999. Responses of Small Mammals and Vegetation to a Reintroduction of Gunnison's Prairie Dogs. Journal of Mammalogy **80**:1311-1324.

Delibes-Mateos, M., E. Ramírez, P. Ferreras, and R. Villafuerte. 2008. Translocations as a risk for the conservation of European wild rabbit Oryctolagus cuniculus lineages. Oryx **42**:259-264.

DeYoung, R. W., E. C. Hellgren, T. E. Fulbright, W. F. Robbins, and I. D. Humphreys. 2000. Modeling nutritional carrying capacity for translocated desert bighorn sheep in western Texas. Restoration Ecology **8**:57-65.

Dimond, W. J. and D. P. Armstrong. 2007. Adaptive Harvesting of Source Populations for Translocation: a Case Study with New Zealand Robins. Conservation Biology **21**:114-124.

Edgar, P. W., R. A. Griffiths, and J. P. Foster. 2005. Evaluation of translocation as a tool for mitigating development threats to great crested newts ( Triturus cristatus) in England, 1990–2001. Biological Conservation **122**:45-52.

Ewing, S. R., R. G. Nager, M. A. C. Nicoll, A. Aumjaud, C. G. Jones, and L. F. Keller. 2008. Inbreeding and Loss of Genetic Variation in a Reintroduced Population of Mauritius Kestrel. Conservation Biology **22**:395-404.

Finlayson, G. R., E. M. Vieira, D. Priddel, R. Wheeler, J. Bentley, and C. R. Dickman. 2008. Multi-scale patterns of habitat use by re-introduced mammals: A case study using medium-sized marsupials. Biological Conservation **141**:320-331.

Garrett, L. J. H., C. G. Jones, A. Cristinacce, and D. J. Bell. 2007. Competition or co-existence of reintroduced, critically endangered Mauritius fodies and invasive Madagascar fodies in lowland Mauritius? Biological Conservation **140**:19-28.

Gibbs, J. P., C. Marquez, and E. J. Sterling. 2008. The Role of Endangered Species Reintroduction in Ecosystem Restoration: Tortoise–Cactus Interactions on Española Island, Galápagos. Restoration Ecology **16**:88-93.

Hicks, J. F., J. L. Rachlow, O. E. Rhodes, C. L. Williams, and L. P. Waits. 2007. Reintroduction and Genetic Structure: Rocky Mountain Elk in Yellowstone and the Western States. Journal of Mammalogy **88**:129-138.

James, A. I. and D. J. Eldridge. 2007. Reintroduction of fossorial native mammals and potential impacts on ecosystem processes in an Australian desert landscape. Biological Conservation **138**:351-359.

Jamieson, I. G. 2004. No evidence that dietary nutrient deficiency is related to poor reproductive success of translocated takahe. Biological Conservation **115**:165-170.

Johnson, T. L. and D. M. Swift. 2000. A test of a habitat evaluation procedure for Rocky Mountain bighorn sheep. Restoration Ecology **8**:47-56.

Kalinowski, S. T., P. W. Hedrick, and P. S. Miller. 1999. No Inbreeding Depression Observed in Mexican and Red Wolf Captive Breeding Programs. Conservation Biology **13**:1371-1377.

Kamler, J. F., R. M. Lee, J. C. deVos, W. B. Ballard, and H. A. Whitlaw. 2002. Survival and Cougar Predation of Translocated Bighorn Sheep in Arizona. The Journal of Wildlife Management **66**:1267-1272.

Kasworm, W. F., M. F. Proctor, C. Servheen, and D. Paetkau. 2007. Success of Grizzly Bear Population Augmentation in Northwest Montana. The Journal of Wildlife Management **71**:1261-1266.

Larson, S., R. Jameson, J. Bodkin, M. Staedler, and P. Bentzen. 2002. Microsatellite DNA and Mitochondrial DNA Variation in Remnant and Translocated Sea Otter (Enhydra lutris) Populations. Journal of Mammalogy **83**:893-906.

Leberg, P. L. and D. L. Ellsworth. 1999. Further Evaluation of the Genetic Consequences of Translocations on Southeastern White-Tailed Deer Populations. The Journal of Wildlife Management **63**:327-334.

Mathews, F., D. Moro, R. Strachan, M. Gelling, and N. Buller. 2006. Health surveillance in wildlife reintroductions. Biological Conservation **131**:338-347.

McTurk, D. and L. Spelman. 2005. Hand‐rearing and rehabilitation of orphaned wild giant otters, Pteronura brasiliensis, on the Rupununi river, Guyana, South America. Zoo Biology **24**:153-167.

Meek, W. R., N. J. Burman, P. J. Burman, M. Nowakowski, and T. H. Sparks. 2003. Barn owl release in lowland southern England—a twenty-one year study. Biological Conservation **109**:271-282.

Meretsky, V. J., S. R. Beissinger, D. A. Clendenen, and J. W. Wiley. 2000. Demography of the California Condor: Implications for Reestablishment. Conservation Biology **14**:957-967.

Munthali, S. M. and F. X. Mkanda. 2002. The plight of Malawi's wildlife: is trans-location of animals the solution? Biodiversity and Conservation **11**:751-768.

Nicoll, M. A. C., C. G. Jones, and K. Norris. 2004. Comparison of survival rates of captive-reared and wild-bred Mauritius kestrels ( Falco punctatus) in a re-introduced population. Biological Conservation **118**:539-548.

Oakleaf, J. K., J. R. Oakleaf, C. C. Niemeyer, D. L. Murray, E. E. Bangs, C. M. Mack, D. W. Smith, J. A. Fontaine, M. D. Jimenez, and T. J. Meier. 2006. Habitat Selection by Recolonizing Wolves in the Northern Rocky Mountains of the United States. The Journal of Wildlife Management **70**:554-563.

Olsson, O., B. Department of, U. Lund, Biodiversitet, Naturvetenskap, Biodiversity, u. Lunds, i. Biologiska, and Science. 2007. Genetic origin and success of reintroduced white storks. Conservation Biology **21**:1196-1206.

Ostro, L. E. T., S. C. Silver, F. W. Koontz, T. P. Young, and R. H. Horwich. 1999. Ranging behavior of translocated and established groups of black howler monkeys Alouatta pigra in Belize, Central America. Biological Conservation **87**:181-190.

Parker, J. M. and S. H. Anderson. 2003. Habitat Use and Movements of Repatriated Wyoming Toads. The Journal of Wildlife Management **67**:439-446.

Perelberg, A., D. Saltz, S. Bar-David, A. Dolev, and Y. Yom-Tov. 2003. Seasonal and Circadian Changes in the Home Ranges of Reintroduced Persian Fallow Deer. The Journal of Wildlife Management **67**:485-492.

Pip, M., D. Toni, B. Steve, and M. Graeme. 2004. Koalas on Kangaroo Island: from introduction to pest status in less than a century. Wildlife Research **31**:267-272.

Pizzuto, T. A., G. R. Finlayson, M. S. Crowther, and C. R. Dickman. 2007. Microhabitat use by the brush-tailed bettong ( Bettongia penicillata ) and burrowing bettong ( B. lesueur ) in semiarid New South Wales: implications for reintroduction programs. Wildlife Research **34**:271-279.

Ramey, R. R., G. Luikart, and F. J. Singer. 2000. Genetic Bottlenecks Resulting from Restoration Efforts: The Case of Bighorn Sheep in Badlands National Park. Restoration Ecology **8**:85-90.

Reindl-Thompson, S. A., J. A. Shivik, and A. Whitelaw. 2006. Efficacy of Scent Dogs in Detecting Black-Footed Ferrets at a Reintroduction Site in South Dakota. Wildlife Society Bulletin [H.W. Wilson - GS] **34**:1435-1439.

Robert, A., F. Sarrazin, D. Couvet, and S. Legendre. 2004. Releasing Adults versus Young in Reintroductions: Interactions between Demography and Genetics. Conservation Biology **18**:1078-1087.

Sarrazin, F. and S. Legendre. 2000. Demographic Approach to Releasing Adults versus Young in Reintroductions. Conservation Biology **14**:488-500.

Schaefer, J. A. and J. Hamr. 2007. Winter Resource Selection of Reintroduced Elk and Sympatric White-Tailed Deer at Multiple Spatial Scales. Journal of Mammalogy **88**:614-624.

Schaub, M., R. Pradel, and J.-D. Lebreton. 2004. Is the reintroduced white stork ( Ciconia ciconia) population in Switzerland self-sustainable? Biological Conservation **119**:105-114.

Seddon, P. J. and K. Ismail. 2002. Influence of ambient temperature on diurnal activity of Arabian oryx: Implications for reintroduction site selection. Oryx **36**:50-55.

Seddon, P. J., K. Ismail, M. Shobrak, S. Ostrowski, and C. Magin. 2003. A comparison of derived population estimate, mark-resighting and distance sampling methods to determine the population size of a desert ungulate, the Arabian oryx. Oryx **37**:286-294.

Sigg, D. P., A. W. Goldizen, and A. R. Pople. 2005. The importance of mating system in translocation programs: reproductive success of released male bridled nailtail wallabies. Biological Conservation **123**:289-300.

Singer, F. J., M. E. Moses, S. Bellew, and W. Sloan. 2000. Correlates to Colonizations of New Patches by Translocated Populations of Bighorn Sheep. Restoration Ecology **8**:66-74.

Singer, F. J., L. C. Zeigenfuss, and L. Spicer. 2001. Role of Patch Size, Disease, and Movement in Rapid Extinction of Bighorn Sheep. Conservation Biology **15**:1347-1354.

Smart, J., A. Amar, I. M. W. Sim, B. Etheridge, D. Cameron, G. Christie, and J. D. Wilson. 2010. Illegal killing slows population recovery of a re-introduced raptor of high conservation concern – The red kite Milvus milvus. Biological Conservation **143**:1278-1286.

Stamatis, C., F. Suchentrunk, H. Sert, C. Triantaphyllidis, and Z. Mamuris. 2007. Genetic evidence for survival of released captive-bred brown hares Lepus europaeus during restocking operations in Greece. Oryx **41**:548-551.

Stephen, C. L., D. G. Whittaker, D. Gillis, L. L. Cox, and O. E. Rhodes. 2005. Genetic Consequences of Reintroductions: An Example from Oregon Pronghorn Antelope (Antilocapra americana). The Journal of Wildlife Management **69**:1463-1474.

Ted, M., J. R. James C. Devos, B. Warren B, and B. O. E. Sue R. 2006. Mountain Lion Predation of Translocated Desert Bighorn Sheep in Arizona. Wildlife Society Bulletin **34**:1255-1263.

Todd, C. R., S. Jenkins, and A. R. Bearlin. 2002. Lessons about extinction and translocation: models for eastern barred bandicoots ( Perameles gunnii) at Woodlands Historic Park, Victoria, Australia. Biological Conservation **106**:211-223.

Tordoff, H. B. and P. T. Redig. 2001. Role of Genetic Background in the Success of Reintroduced Peregrine Falcons. Conservation Biology **15**:528-532.

van Heezik, Y., K. Ismail, and P. J. Seddon. 2003. Shifting spatial distributions of Arabian oryx in relation to sporadic water provision and artificial shade. Oryx **37**:295-304.

Vinkey, R. S., M. K. Schwartz, and K. S. McKelvey. 2006. When Reintroductions Are Augmentations: the Genetic Legacy of Fishers (Martes pennanti) in Montana. Journal of Mammalogy [H.W. Wilson - GS] **87**:265.

White, P. C. L., C. J. McClean, and G. L. Woodroffe. 2003. Factors affecting the success of an otter ( Lutra lutra) reinforcement programme, as identified by post-translocation monitoring. Biological Conservation **112**:363-371.

Whittaker, D. G., S. D. Ostermann, and W. M. Boyce. 2004. Genetic Variability of Reintroduced California Bighorn Sheep in Oregon. The Journal of Wildlife Management **68**:850-859.

Williams, R. N., O. E. Rhodes, and T. L. Serfass. 2000. Assessment of Genetic Variance among Source and Reintroduced Fisher Populations. Journal of Mammalogy **81**:895-907.

Wisely, S. M., D. B. McDonald, and S. W. Buskirk. 2003. Evaluation of the genetic management of the endangered black‐footed ferret (Mustela nigripes). Zoo Biology **22**:287-298.

Zeigenfuss, L. C., F. J. Singer, and M. A. Gudorf. 2000. Test of a Modified Habitat Suitability Model for Bighorn Sheep. Restoration Ecology **8**:38-46.

**Supplementary material references**

Armstrong, D. P. and P. J. Seddon. 2008. Directions in reintroduction biology. Trends in Ecology & Evolution **23**:20-25.

Bain, D. and K. French. 2009. Impacts on a threatened bird population of removals for translocation. Wildlife Research **36**:516-521.

Cocks, L. and K. Bullo. 2008. The processes for releasing a zoo‐bred Sumatran orang‐utan Pongo abelii at Bukit Tigapuluh National Park, Jambi, Sumatra. International Zoo Yearbook **42**:183-189.

Eastridge, R. and J. D. Clark. 2001. Evaluation of 2 Soft-Release Techniques to Reintroduce Black Bears. Wildlife Society Bulletin **29**:1163-1174.

Fischer, J. and D. Lindenmayer. 2000. An assessment of the published results of animal relocations. Biological Conservation **96**:1-11.

Frair, J. L., E. H. Merrill, and J. R. Allen. 2007. Know Thy Enemy: Experience Affects Elk Translocation Success in Risky Landscapes. The Journal of Wildlife Management [H.W. Wilson - GS] **71**:541.

Griffith, B., J. M. Scott, J. W. Carpenter, and C. Reed. 1989. Translocation as a species conservation tool: status and strategy. Science(Washington) **245**:477-480.

Hamilton, L. P., P. A. Kelly, D. F. Williams, D. A. Kelt, and H. U. Wittmer. 2010. Factors associated with survival of reintroduced riparian brush rabbits in California. Biological Conservation **143**:999-1007.

Harding, L. E., O. F. Abu-Eid, N. Hamidan, and A. al Sha'lan. 2007. Reintroduction of the Arabian oryx Oryx leucoryx in Jordan: war and redemption. Oryx **41**:478-487.

Hu, H. and Z. Jiang. 2002. Trial release of Père David's deer Elaphurus davidianus in the Dafeng Reserve, China. Oryx **36**:196-199.

Hughes, J. B., G. C. Daily, and P. R. Ehrlich. 1997. Population diversity: its extent and extinction. Science **278**:689-692.

Hunter, L. T. B., K. Pretorius, L. C. Carlisle, M. Rickelton, C. Walker, R. Slotow, and J. D. Skinner. 2007. Restoring lions Panthera leo to northern KwaZulu-Natal, South Africa: short-term biological and technical success but equivocal long-term conservation. Oryx **41**:196-204.

Massei, G., R. J. Quy, J. Gurney, and D. P. Cowan. 2010. Can translocations be used to mitigate human–wildlife conflicts? Wildlife Research **37**:428-439.

Nakagawa, S. and I. C. Cuthill. 2007. Effect size, confidence interval and statistical significance: a practical guide for biologists. Biological Reviews **82**:591-605.

Priddel, D., N. Carlile, and R. Wheeler. 2006. Establishment of a new breeding colony of Gould’s petrel ( Pterodroma leucoptera leucoptera) through the creation of artificial nesting habitat and the translocation of nestlings. Biological Conservation **128**:553-563.

Reindl-Thompson, S. A., J. A. Shivik, and A. Whitelaw. 2006. Efficacy of Scent Dogs in Detecting Black-Footed Ferrets at a Reintroduction Site in South Dakota. Wildlife Society Bulletin [H.W. Wilson - GS] **34**:1435-1439.

Seddon, P. J., D. P. Armstrong, and R. F. Maloney. 2007. Developing the science of reintroduction biology. Conservation Biology **21**:303-312.

Sheean, V. A., A. D. Manning, and D. B. Lindenmayer. 2012. An assessment of scientific approaches towards species relocations in Australia. Austral Ecology **37**:204-215.

Shier, D. M. 2006. Effect of Family Support on the Success of Translocated Black-Tailed Prairie Dogs. Conservation Biology **20**:1780-1790.

Shier, D. M. and D. H. Owings. 2006. Effects of predator training on behavior and post-release survival of captive prairie dogs ( Cynomys ludovicianus). Biological Conservation **132**:126-135.

Williams, C. K., G. Ericsson, and T. A. Heberlein. 2002. A Quantitative Summary of Attitudes toward Wolves and Their Reintroduction (1972-2000). Wildlife Society Bulletin **30**:575-584.
